# Real-world practice of conversion surgery for unresectable hepatocellular carcinoma - a single center data of 26 consecutive patients

**DOI:** 10.1186/s12885-023-10955-7

**Published:** 2023-05-20

**Authors:** Bo Zhang, Xuetao Shi, Kai Cui, Zhongchao Li, Lei Li, Zhaogang Liu, Chengsheng Zhang, Pengfei Sun, Jingtao Zhong, Zhicheng Sun, Zhibin Chang, Zhao Ma, Alex Gordon-Weeks, Mingming Li, Lei Zhao

**Affiliations:** 1 Department of Hepatobiliary Surgery, Shandong Cancer Hospital Affiliated to Shandong First Medical University, 440 Jiyan Road, Huaiyin District, Jinan, 250117 China; 2 Shandong First Medical University, 6699 Qingdao Road, Huaiyin District, Jinan, 250117 China; 3 The Fourth People’s Hospital of Jinan, Jinan, 250031 China; 4 Nuffield Department of Surgical Sciences, University of Oxford, Oxford, OX3 9DU United Kingdom

**Keywords:** Hepatocellular carcinoma, Conversion therapy, Tumor response, Treatment-related adverse events (TRAEs)

## Abstract

**Aim:**

To understand the proportion of uHCC (unresectable hepatocellular carcinoma) patients who achieve successful conversion resection in a high-volume setting with state of the art treatment options.

**Methods:**

We retrospectively reviewed all HCC patients hospitalized to our center from June 1^st^, 2019 to June 1^st^, 2022. Conversion rate, clinicopathological features, response to systemic and/or loco-regional therapy and surgical outcomes were analyzed.

**Results:**

A total of 1,904 HCC patients were identified, with 1672 patients receiving anti-HCC treatment. 328 patients were considered up-front resectable. Of the remaining 1344 uHCC patients, 311 received loco-regional treatment, 224 received systemic treatment, and the remainder (809) received combination systemic plus loco-regional treatment. Following treatment, one patient from the systemic group and 25 patients from the combination group were considered to have resectable disease. A high objective response rate (ORR) was observed in these converted patients (42.3% under RECIST v1.1 and 76.9% under mRECIST criteria). The disease control rate (DCR) reached 100%. 23 patients underwent curative hepatectomy. Major post-operative morbidity was equivalent in the both groups (*P*=0.76). Pathologic complete response (pCR) was 39.1%. During conversion treatment, grade 3 or higher treatment-related adverse events (TRAEs) were observed in 50% of patients. The median follow-up time was 12.9 months (range, 3.9~40.6) from index diagnosis and 11.4 months (range, 0.9~26.9) from resection. Three patients experienced disease recurrence following conversion surgery.

**Conclusions:**

By intensive treatment, a small sub-group of uHCC patients (2%) may potentially be converted to curative resection. Loco-regional combined with systemic modality was relative safe and effective in the conversion therapy. Short-term outcomes are encouraging, but long-term follow-up in a larger patient population are required to fully understand the utility of this approach.

**Supplementary Information:**

The online version contains supplementary material available at 10.1186/s12885-023-10955-7.

## Introduction

Primary liver cancer (PLC) is increasingly prevalent worldwide, the sixth most commonly diagnosed cancer and the third leading cause of cancer death. The annual new cases of liver cancer are estimated to increase by 50% between 2020 and 2040, and 1.3 million patients are predicted to die from PLC per year by 2040 [1]. Hepatocellular carcinoma (HCC) represents approximately 75% to 85% of PLCs [2]. Due to the lack of effective non-surgical treatments, the outcomes of HCC used to be very dismal.

In the last decade, the development of targeted therapies and immune checkpoint inhibitors (ICIs), has contributed to improved survival in a subset of unresectable hepatocellular carcinoma (uHCC) patients. In the IMbrave 150 study, combination atezolizumab and bevacizumab significantly increased median overall survival (mOS) to 19.2 months [3]; whilst the LEAP002 study, although the combination of Lenvatinib and pembrolizumab failed to show the significant superiority to Lenvatinib alone, the median overall survival (mOS) reached 21.2 months and 19.0 months, respectively [4]. Despite this progress, longer-term survival such that oncological cure can be claimed in initially uHCC patients is rarely reported. This is probably because this type of trial has focused on the effect of the systemic therapies, and not included the possible following surgery in the standard protocol. In the IMbrave 150 study, only 5 (1.5%) patients in the combination arm received follow-up surgical therapy after disease progression (The appendix to [5]).

Radical liver-directed approaches (surgical resection, transplantation or ablation) remain the only curative-intent options for HCC, with 5-year survival rate over 60% in patients who meet select criteria. But, at diagnosis, only 10-30% of HCC patients are candidates for liver-directed therapy [6]. Conversion therapy and methods to increase the proportion of patients who experience successful conversion therefore has the potential to significantly increase long-term survival and cure in HCC patients.

In the “Chinese expert consensus on conversion therapy for hepatocellular carcinoma (2021 edition)”, conversion therapy is defined as the “conversion of an unresectable HCC into resectable HCC followed by surgical removal of the tumor” [7].

In 2021, Zhu et al. first reported a cohort study of conversion therapy in uHCC by tyrosine kinase inhibitors (TKIs) plus anti-PD-1, in a group of sixty-three consecutive patients, with 10 (15.9%) ultimately achieving R0 resection [8]. Subsequent results from a larger patient cohort in the same centre achieved complete conversion in 24% of patients with initial uHCC [9]. Zhang et al. reported a smaller cohort of patients experiencing conversion therapy through combination TKIs and anti-PD-1 [10]. More recently, cohort study of the conversion therapies by the combinations of toripalimab (an anti-PD-1), lenvatinib plus transcatheter arterial chemoembolization (TACE) [11], as well as the combinations of anti-PD-1, lenvatinib and hepatic artery infusion chemotherapy (HAIC) [12] have been reported.

These studies demonstrate the efficacy of conversion therapies with various modalities, while in the scenario of the real-world practice, the treatment modalities of uHCC are not uniform but heterogeneous and varied. Here we report the real-world data of consecutive 26 cases of uHCC conversion therapy in our center.

## Materials and methods

### Patients

From June 1^st^, 2019 to June 1^st^, 2022, consecutive HCC patients at our center (“the Department of Hepatobiliary Surgery of Shandong Cancer Hospital Affiliated to Shandong First Medical University”) were retrospectively reviewed. The diagnosis of HCC were based on the AASLD guideline [13] and the “Guidelines For The Diagnosis And Treatment Of Primary Liver Cancer” by the Chinese Ministry of Health [14]. The treatments of all patients were discussed in a multidisciplinary team (MDT) meeting consisting of hepatologists, liver surgeons, medical oncologists, radiation oncologists, interventional radiologists, pathologists and radiologists with expertise covering all potential treatment options. For surgically-treated patients (upfront or conversion surgeries), diagnoses were confirmed through pathologic examination. Those who were diagnosed as uHCC and receive systemic and/or loco-regional therapies, then following by the curative resection were defined as patients of conversion surgery, their clinicopathological features, responses to the systemic and/or loco-regional treatments, safety and the preliminary outcomes of conversion surgery and conversion rate were analyzed.

Patients were considered initially unresectable if they met any of the following criteria: extra-hepatic metastasis (EHM), major venous tumor thrombosis or giant solitary tumor with insufficient future liver remnant (FLR). The exact reasons of unresectability of each patient are presented in the “ Results”.

All research in this study was conducted in accordance with both the Declarations of Helsinki and Istanbul. The study was approved by the Ethics Committee of Shandong Cancer Hospital Affiliated to Shandong First Medical University, with waiver of informed consent. There are three reasons. First, the period of this study is long. Second, no patient privacy information is involved. Third, this is a retrospective study, it did not intervene the decisions of the treatments.

### Treatments

For systemic therapies, a number of regimens were used according to the international and local practice guidelines. The TKIs used in this study were lenvatinib (8mg/day), apatinib (250 mg/day) or sorafenib (800mg/day). Anti PD-1 antibodies were intravenously administered as follows: sintilimab 200 mg, or camrelizumab 200 mg, every 3 to 4 weeks.

TACE treatment was carried out according to the standard procedure. Formal angiographic assessment was performed in all patients in whom TACE was felt appropriate. In conventional TACE (cTACE), a mixture of lipiodol plus epirubicin was injected followed by embolic agents (gelatin sponge particles or microspheres), while in dTACE, microspheres (Callispheres®) loaded with epirubicin 80mg/g were used with or without supplementary particles.

Hepatic artery infusion chemotherapy (HAIC) treatment was carried out according to the reported procedure [15] and divided into 3-week cycles. If tumor also got arterial feeding from the right inferior phrenic artery or other collateral vessels, the collateral vessels were to be embolized to achieve blood flow redistribution, thus ensuring the efficiency of following drug infusion.

Patients were then transferred back to the inpatient ward and received drug infusion via the hepatic artery: oxaliplatin, 130 mg/m^2^ from hour 0-2 on day 1; leucovorin, 400 mg/m^2^ from hour 2-3 on day 1; and fluorouracil, 400 mg/m^2^ bolus at hour 3 on day 1 and 2,400 mg/m^2^ over 24 or 46 hours. After HAIC was completed, the catheter and sheath were removed. Repetitive femoral artery puncture and catheterization were performed in the next HAIC cycle to re-evaluate the blood supply of the tumor and adjusted the superselective location if necessary.

### Response and toxicity evaluation

All patients were monitored and evaluated regularly. Briefly, lab tests (complete blood count, liver, renal, thyroid and cardiac functions, and tumor markers) were repeated before each treatment cycle. Tumor response and resectability were evaluated via contrast-enhanced magnetic resonance imaging/computed tomography (MRI/CT) at least every 2 treatment cycles by the multidisciplinary team of hepatobiliary tumors. Tumor responses were graded as complete response (CR), partial response (PR), stable disease (SD), or progressive disease (PD) according to RECIST v1.1 and modified RECIST (mRECIST) criteria. The objective response rate (ORR) was calculated as the sum of CR and PR. The disease control rate (DCR) was calculated as the sum of CR, PR, and SD. Adverse events were assessed and graded using the National Cancer Institute Common Terminology Criteria for Adverse Events v4.0, immune-related AEs (irAEs) were also assessed.

To evaluate the safety of conversion surgery, serial perioperative data, including the changes of the percentages of blood and liver test parameters (ΔRBC%, ΔHb%, ΔALT%, ΔAST%, Δbilirubin%, Δalbumin%), the operation time, the mean blood loss, the postoperative hospital stay and the drainage time, as well as the incidences of the postoperative complications, including infection, bile leakage, hypoproteinemia, significant ascites, hydrothorax, etc., were collected.

OS was defined using the time from initial diagnosis, and from the date of surgery. Recurrence-free survival was defined as the interval between the date of surgery and the date of recurrence.

### Postoperative management

TKIs and/or anti-PD-1 antibodies were resumed approximately 4 weeks after hepatectomy when patients were fully recovered from surgery. Postoperative radiographic (contrast enhanced abdominal CT or MRI) as well as biochemical (AFP) assessments were performed at least every 3 months in the first year and at least every 6 months in the following year. Adjuvant therapy was discontinued 12 months after surgery, following MDT discussion as well as the consideration of patients’ preference. For recurrent patients, the treatment modalities were based on the pattern of tumor recurrence and MDT suggestion.

### Statistical analysis

Statistical analysis was performed using Statistical Package for the Social Sciences (SPSS, version 17.0; SPSS Inc., Chicago, IL, USA). Quantitative variables are presented as the mean ± standard deviation. Student’s t test or Mann–Whitney U test was performed to compare differences between groups. Chi‑square (χ2) test was used to assess the ECOG PS scores and post-operative complications in different groups. Categorical data was expressed as frequencies (percentages). The association between successful conversion and those baseline stratification factors were analysed by Cox proportional-hazards model to generate HRs and 95% CIs.

## Results

### Patient characteristics

**Fig. 1:**
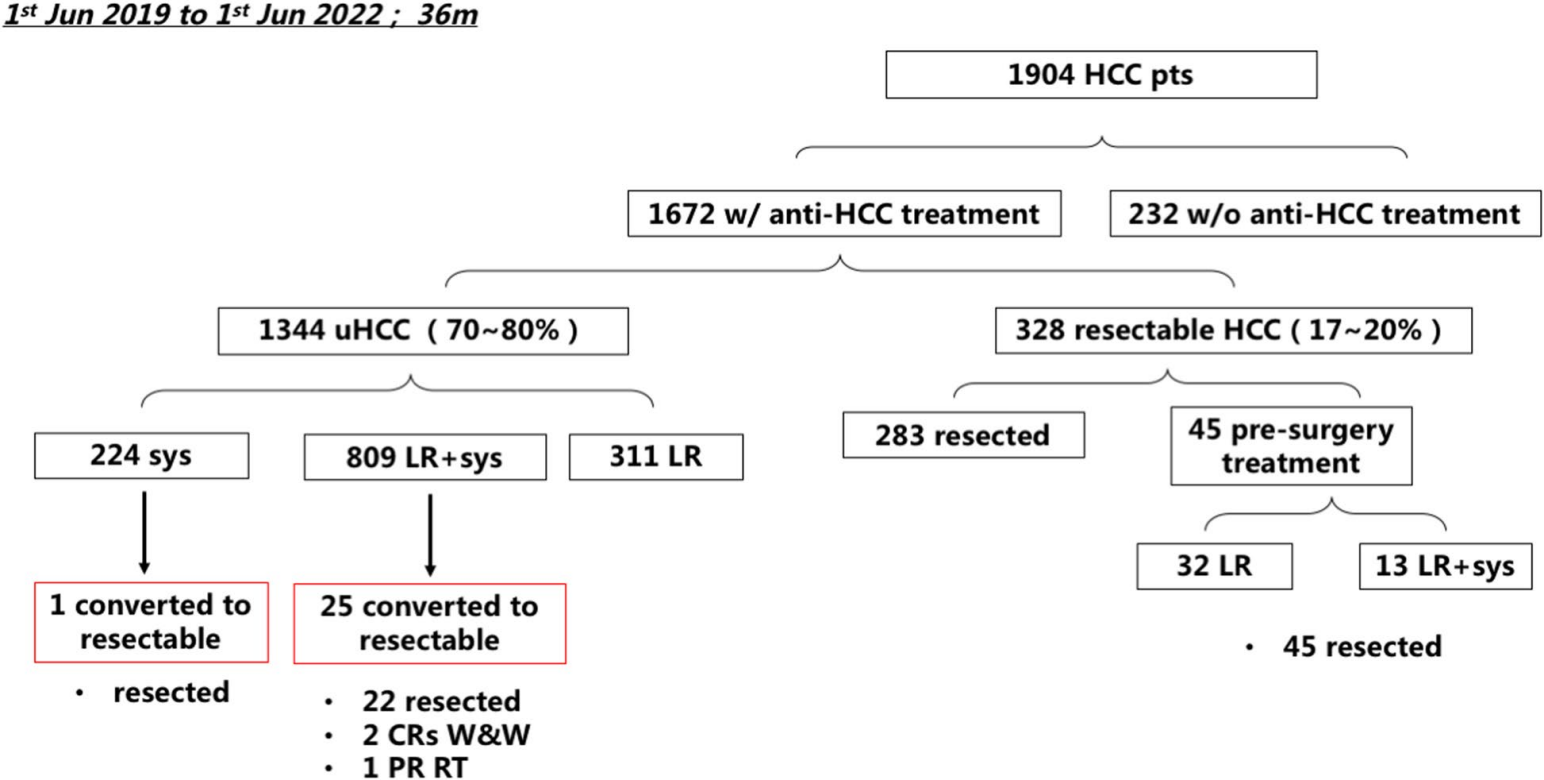
Patient flow. From June 1st 2019 to June 1st 2022, total numbers of HCC patients admitted to our center, numbers of patients received or did not received anti-HCC treatment, numbers of patients with initially resectable or unresectable HCC (uHCC), numbers of uHCC patients received different treatment modalities, as well as the numbers of converted patients are presented. HCC, hepatocellular carcinoma; pts, patients; w/, with; w/o, without; uHCC, unresectable hepatocellular carcinoma; sys, systemic treatment; LR, loco-regional therapy; CR, complete response; W&W, watch & wait; PR, partial response; RT, radiotherapy

Patient flow is demonstrated in Fig. 1. From June 1^st^ 2019 to June 1^st^ 2022, a total of 1,904 HCC patients were admitted to our center, 232 of them did not receive any anti-cancer treatment, while other 1672 patients received different anti-HCC treatments. Following combinations of targeted, ICI and/or loco-regional therapy, 26 patients were considered suitable for surgical resection with curative intent.

The median age of the 26 converted patients was 56.4 years (range: 28 to 76 years); All patients had hepatitis B virus (HBV) infection, 16 patients (61.5%) had AFP over 400 ng/ml, 25 patients (96.2%) had Child-Pugh A liver function; the tumors of 16 patients (61.5%) were solitary; 19 patients (73.1%) were Barcelona Clinic Liver Cancer (BCLC) C stage. Their status at the outset before treatment started were summarized in Table 1; the stage and exact reason(s) for being initially unresectable of every patient were summarized in Table 2.

**Table 1:** Baseline characteristics of 26 converted HCC patients

**Characteristics**	**Patients (*n*=26)**
**Median age, years (range)**	56.4 (28~76)
**Age, years, *n*** ** (%)**	
＜65	19 (73.1)
≥65	7 (26.9)
**Sex, *n* (%)**	
Female	4 (15.4)
Male	22 (84.6)
**ECOG PS, *n*** ** (%)**	
0	10 (38.5)
1	15 (57.7)
2	1 (3.8)
**Hepatitis B virus infection, *n* (%)**	26 (100)
**Serum AFP level,* n* (%)**	
<400ng/ml	10 (38.5)
400-1000ng/ml	1 (3.8)
≥1000ng/ml	15 (57.7)
**Tumor number, *n* (%)**	
Solitary	16 (61.5)
Multiple	10 (38.5)
**Tumor size, *n* (%)**	
＜10cm	12 (46.2)
≥10cm	14 (53.8)
**Child-Pugh classification, *n* (%)**	
A	25 (96.2)
B	1 (3.8)
**BCLC staging, *n* (%)**	
A	6 (23.1)
B	1 (3.8)
C	19 (73.1)
**CNLC staging, *n* (%)**	
I	6 (23.1)
II	1 (3.8)
III	19 (73.1)
**Liver cirrhosis, *n* (%)**	21 (80.8)
**Hilar lymphatic metastasis, *n* (%)**	3 (11.5)
**Intrahepatic metastasis, *n* (%)**	2 (7.7)
**Extrahepatic metastasis, *n* (%)**	2 (7.7)
**Reasons of unresectability, *n* (%)**	
Vp2	1 (3.8)
Vp3	10 (38.5)
Vp4	3 (11.5)
Vv2	1 (3.8)
Vv3	4 (15.4)
Insufficient FLR	11 (42.3)
EHM	5 (19.2)

*CNLC* China liver cancer staging, *BCLC* Barcelona Clinic Liver Cancer, *Vp2* Invasion of (or tumor thrombus in) second order branches of the portal vein, *Vp3* Invasion of (or tumor thrombus in) first order branches of the portal vein, *Vp4* Invasion of (or tumor thrombus in) the main trunk of the portal vein and/or contra-lateral portal vein branch to the primarily involved lobe, *Vv2* Invasion of (or tumor thrombus in) the right middle, or left hepatic vein, the inferior right hepatic vein, or the short hepatic vein, *Vv3* Invasion of (or tumor thrombus in) the inferior vena cava, *EHM* Extrahepatic metastasis, *FLR* future liver remnant

**Table 2:** The stage and reasons of unresectability of all patients (*n*=26)

**Patients**	**CNLC stage**	**BCLC stage**	**Reasons of unresectability**
1	Ib	A	Insufficient FLR
2	Ib	A	Insufficient FLR
3	IIIa	C	Vp4
4	IIIb	C	Vp3, EHM
5	Ib	A	Insufficient FLR
6	IIIa	C	Vp3
7	IIIb	C	EHM
8	IIb	B	Insufficient FLR
9	IIIa	C	Vp3
10	IIIa	C	Vv3
11	IIIa	C	Vp2, Insufficient FLR
12	IIIa	C	Vp3, Insufficient FLR
13	IIIa	C	Vv2
14	IIIa	C	Vp3, Insufficient FLR
15	IIIa	C	Vp3
16	Ib	A	Insufficient FLR
17	IIIb	C	Vp3, Vv3, EHM
18	IIIa	C	Vp4
19	Ib	A	Insufficient FLR
20	IIIb	C	EHM
21	IIIa	C	Vp3, Vv3
22	Ib	A	Insufficient FLR
23	IIIa	C	Vp3, Vv3
24	IIIa	C	Vp4
25	IIIb	C	EHM
26	IIIa	C	Vp3, Insufficient FLR

*Vp2* Invasion of (or tumor thrombus in) second order branches of the portal vein, *Vp3* Invasion of (or tumor thrombus in) first order branches of the portal vein, *Vp4* Invasion of (or tumor thrombus in) the main trunk of the portal vein and/or contra-lateral portal vein branch to the primarily involved lobe, *Vv2* Invasion of (or tumor thrombus in) the right middle, or left hepatic vein, the inferior right hepatic vein, or the short hepatic vein, *Vv3* Invasion of (or tumor thrombus in) the inferior vena cava, *EHM* Extrahepatic metastasis, *FLR* Future liver remnant

### Tumor response

Treatment responses of 26 converted patients were summarized in Table 3. Under the RECIST v1.1 criteria, 11 patients (42.3%) reached PR, no CR was observed; all other 15 patients (57.7%) were classified as SD, so the DCR reached 100%. Under mRECIST criteria, 5 patients (19.2%) achieved CR, 15 patients (57.7%) were evaluated as PR, so the ORR reached 76.9%; the DCR was also 100%. No patient experienced PD. After conversion, twenty-three patients (88.5%) underwent R0 resection, in whom pCR was found in 9 of 23 specimens (39.1%). There were 3 patients (2 CRs and 1 PR) chose “W&W” (watch and wait) policy and did not undergo surgical resection. The tumor response of different treatment modalities is summarized in Table 4.

**Table 3:** Assessment of objective response by RECIST and mRECIST criteria

Variable, *n* (%)	*n*=26	
	RECIST v1.1	mRECIST
Complete response, *n* (%)	0 (0)	5 (19.2)
Partial response, *n* (%)	11 (42.3)	15 (57.7)
Objective response, *n* (%)	11 (42.3)	20 (76.9)
Stable disease, *n* (%)	15 (57.7)	6 (23.1)
Disease control rate, *n* (%)	26 (100)	26 (100)
Progressive disease, *n* (%)	0(0)	0(0)
R0 resection, *n* (%)	23 (88.5)	
Pathologic complete response,* n* (%)	9 (39.1)	

*mRECIST* Modified RECIST criteria, *CR* Complete response, *PR* Partial response, *SD* Stable disease, *PD* Progressive disease, *pCR* Pathologic complete response, *ORR* Objective response, *DCR* disease control rate

**Table 4:** Tumor response of different treatment modalities (*n*=26)

Treatment modalities	Patients (*n*, %)	ORR (%)^a^	ORR (%)^b^
TKI+PD-1/PD-L1 monoclonal antibody	1 (3.8)	1 (100)	1 (100)
Sorafenib + Sintilimab	1	1 (100)	1 (100)
Locoregional therapies + TKI	4 (15.4)	1 (25)	3 (75)
TACE + Lenvatinib	3	0 (0)	1 (33.3)
TACE + Radiotherapy+ Lenvatinib	1	0 (0)	1 (100)
Locoregional therapies +TKI + Anti-PD-1	21 (80.8)	10 (47.6)	17 (81)
HAIC + Lenvatinib + Camrelizumab	12	5 (41.7)	8 (66.7)
HAIC + Apatinib + Camrelizumab	3	2 (66.7)	3 (100)
TACE + Sorafenib +Sintilimab	1	0 (0)	1 (100)
TACE + Sorafenib + Camrelizumab	2	2 (100)	2 (100)
TACE + Lenvatinib + Camrelizumab	1	0 (0)	1 (100)
TACE + Apatinib + Camrelizumab	2	1 (50)	2 (100)

^a^RECIST v1.1

^b^mRECIST

There were 5 patients of BCLC stage C with oligo-EHM, the exact metastasized sites were: 3 with portal lymph node metastases, the other two with a solitary metastasis to the right lung and the bone, respectively. After conversion therapy, the metastatic lesions of all 5 patients responded well to the treatment: in 3 portal lymph node metastases, 1 was evaluated as CR and 2 as PR by MRI, and no portal lymph node metastasis were found during the operation and the following pathologic examination. The bone metastasis was evaluated as CR by bone scintigraphy, and the lung metastasis was evaluated as SD by CT scan, and both were proved negative by PET-CT during the post-operative follow-up.

**Fig. 2:**
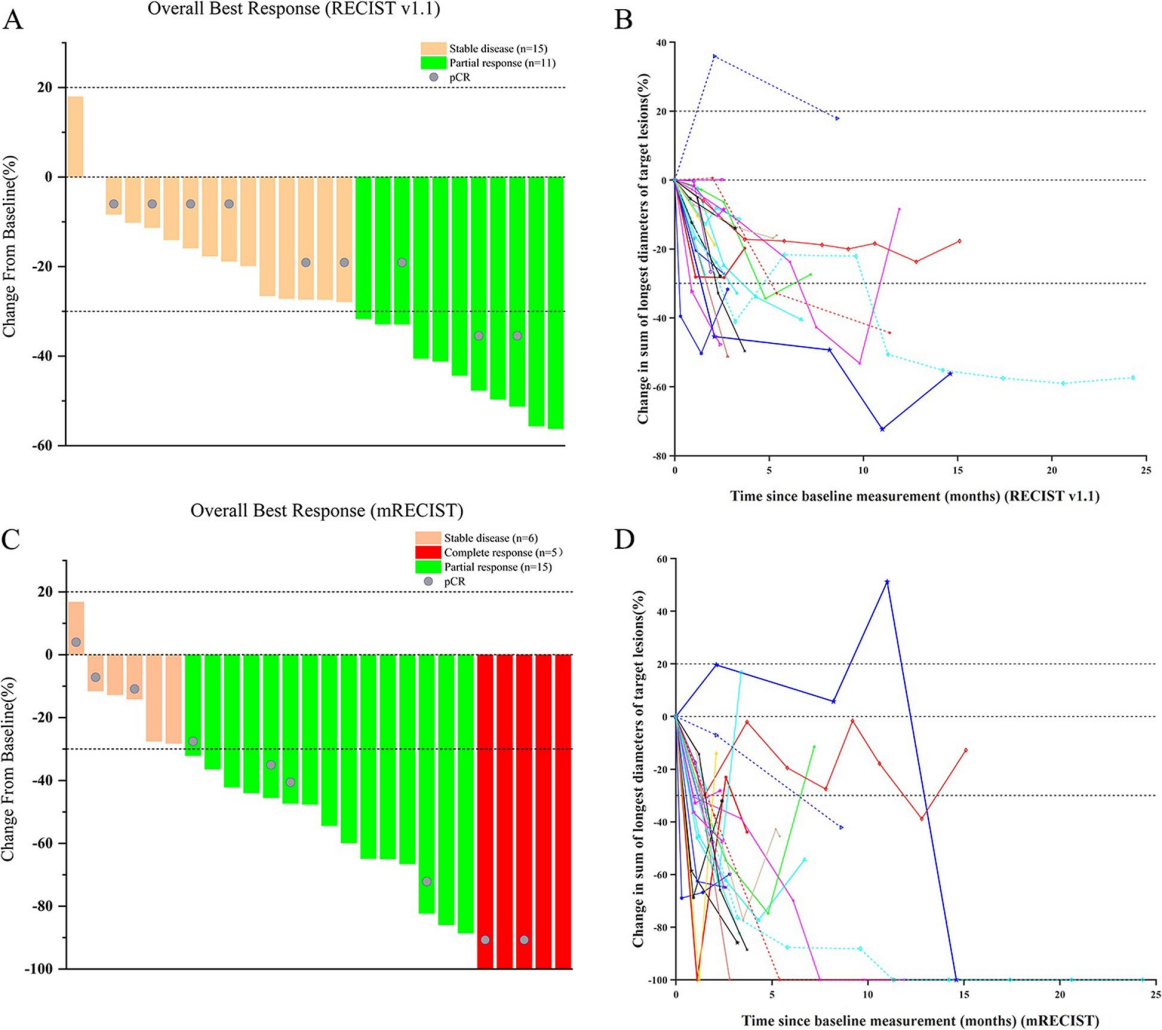
Tumor responses in the conversion therapy. **A** Overall best responses of 26 patients under the RECIST 1.1 criteria. **B** Dynamic tumor responses of 26 patients at every evaluation under the RECIST 1.1 criteria. **C** Overall best responses of 26 patients under the mRECIST criteria. **D** Dynamic tumor responses of 26 patients at every evaluation under the mRECIST criteria

Waterfall plots displaying the optimal tumor response, and the line charts showed the dynamic tumor response from every evaluation under RECIST v 1.1 (A, B) and mRECIST (C, D) are presented in Fig. 2.

### Safety of the conversion therapies

During the conversion treatments, treatment-related adverse events (TRAEs) of any grade occurred in 25 of 26 patients (96.2%); TRAEs over grade 3 occurred in 13 patients (50.0%). The most frequently-occurred TRAEs of both any grade and high grade were impaired liver functions, indicated by the elevation of ALT, AST and bilirubin. Most of the AEs were mild and curative after treatment, no fatal AEs was reported. The only case of grade 4 TRAE (1/26, 3.8%) was irAE (ICI–associated myocarditis). The patient recovered after high-dose methylprednisolone treatment, and ceased the PD-1; he continued to take lenvatinib after recovery and achieved major PR. Ultimately no patients were withdrawn from treatment. The incidences of TRAEs were presented in Table 5.

**Table 5:** TRAEs in 26 converted patients

TRAEs	Any grade (*n*, %)	Grade1~2 (*n*, %)	Grade≥3 (*n*, %)
Hypertension	1 (3.8)	0 (0)	1 (3.8)
Decreased appetite	3 (11.5)	3 (11.5)	0 (0)
Abdominal pain	3 (11.5)	3 (11.5)	0 (0)
Nausea	2 (7.7)	2 (7.7)	0 (0)
Elevated alanine aminotransferase, ALT	16 (61.5)	12 (46.2)	4 (15.4)
Elevated aspartate aminotransferase, AST	19 (73.1)	13 (50)	6 (23.1)
Elevated blood bilirubin	13 (50)	12 (46.2)	1 (3.8)
Hypothyroidism	3 (11.5)	3 (11.5)	0 (0)
Palmar-plantar erythrodyses-thesia syndrome	2 (7.7)	2 (7.7)	0 (0)
Fatigue	1 (3.8)	1 (3.8)	0 (0)
Low leucocyte amount	3 (11.5)	2 (7.7)	1 (3.8)
Neutrocytopenia	1 (3.8)	1 (3.8)	0 (0)
Thrombocytopenia	7 (26.9)	5 (19.2)	2 (7.7)
Oral mucositis	1 (3.8)	0 (0)	1 (3.8)
Rash	1 (3.8)	1 (3.8)	0 (0)
ICIs-associated myocarditis	1 (3.8)	0 (0)	1 (3.8)
RCCEP	1 (3.8)	1 (3.8)	0 (0)

*TRAEs* Treatment-related adverse events, *ICIs* Immune checkpoint inhibitors, *RCCEP* Reactive cutaneous capillary endothelial proliferation

### Safety of the conversion surgery

Compared to 283 R0-resectable patients who received up-front resection, the conversion surgery group of 23 patients showed significantly longer operation time (229.2±59.3 *vs*. 189.01±62.71; min, mean±SD; *P*<0.01), more blood loss (163±152.4 *vs*. 108.29±87.97; ml, mean±SD; *P*<0.01), longer postoperative hospital stay (11.4±3.1 *vs*. 9.98±3.48; days, mean±SD; *P*=0.05) and longer abdominal drainage time (7.91±3.74 *vs*. 5.99±4.33; days, mean±SD; *P*=0.04). In the conversion surgery group, 69.6% (16 out of 23 patients) were of BCLC C stage, while only 21 out of 283 patients (7.4%) in the direct resection group were of BCLC C stage (*P*<0.01) (Table 6). When comparing only the patients of BCLC C stage in the up-front resected and conversion groups, there was no statistic differences in the operation time, mean blood loss, the hospital stay, whilst abdominal drainage time in the conversion group remained significantly longer (8.53±3.62 *vs*. 5.9±2.99, days, mean±SD; *P*=0.03) (Table 7). There was no significant difference between the two groups in post-operative complication.

**Table 6:** Comparison of perioperative conditions in the conversion treatment group and the direct surgery group

	**Conversion surgery (*n*=23)**	**Direct surgery (*n*=283)**	**t/Z/χ2**	***P* value**
**Age, years, *n***			0.25	0.62
＜65	17	228		
≥65	6	55		
**Sex, *n***			0.01	0.61
Male	20	244		
Female	3	39		
**BCLC staging, *n***			71.55	**<0.01**
0~B	7	262		
C	16	21		
**The operation time, min**	229.2±59.3	189.01±62.71	2.97	**<0.01**
**The mean blood loss, ml**	163±152.4	108.29±87.97	-2.62	**<0.01**
**The postoperative hospital stay, days**	11.4±3.1	9.98±3.48	1.95	**0.05**
**The abdominal drainage time, days**	7.91±3.74	5.99±4.33	2.03	**0.04**
**ΔRBC%**	-8.6 (-17.4~-3.1)	-7.2 (-12.6~-1.2)	-1.42	0.16
**ΔHB%**	-8.3 (-16.3~-1.2)	-6.71 (-11.9~-1.4)	-1.02	0.31
**ΔALT%**	956.3 (374.1~1591.7)	688.02 (346.79~1159.7)	-1.57	0.12
**ΔAST%**	864.6 (425~1830.1)	666.35 (353.1~1099.6)	-1.46	0.14
**Δ Bilirubin%**	67.7 (29.3~90.6)	57.78 (15.8~109.6)	-0.59	0.55
**Δ Albumin%**	-14 (-18.2~-6.1)	-15.57 (-22.78~-7.35)	-0.86	0.39
**Postoperative complications**	16 (*n*=13)	266 (*n*=169)	0.09	0.76
Fever caused by infection	2	48		
Biliary leakage	1	1		
Hypoproteinemia	2	77		
Massive ascites	2	60		
Spontaneous bacterial peritonitis	2	4		
Hydrothorax	1	50		
Kaliopenia	5	3		
Incision seepage	1	1		
DVT	0	4		
Pneumonia	0	14		
Pelvic effusion	0	4		

*DVT* Deep venous thrombosis

**Table 7:** Baseline characteristics and surgical outcomes in the patients of BCLC C stage

	**Conversion treatment group (*n*=16)**	**Control group (*n*=21)**	**t/Z/χ2**	***P* value**
**Age, years, *n***			0.03	0.57
< 65	11	15		
≥65	5	6		
**Sex, *n***			0.13	0.6
Male	15	19		
Female	1	2		
**The operation time, min**	236.06±63.83	195±63.72	1.94	0.06
**The mean blood loss, ml**	146.88±64.47	113.5±56.31	-1.47	0.14
**The postoperative hospital stay, days**	11.69±2.75	10.14±3.77	1.38	0.18
**The abdominal drainage time, days**	8.53±3.62	5.9±2.99	2.36	**0.03**
**ΔRBC%**	-8.6 (-17.4~-3.1)	-7.2 (-12.6~-1.2)	-1.42	0.16
**ΔHB%**	-8.3 (-16.3~-1.2)	-6.71 (-11.9~-1.4)	-1.02	0.31
**ΔALT%**	881.46(366.94~1820.15)	743.31(350.19~1263.12)	-0.89	0.37
**ΔAST%**	832.52(380.45~2341.99)	655.51(373.75~1088.99)	-0.64	0.52
**Δ Bilirubin%**	69.78 (34.81~120.02)	34.62 (-1.74~94.28)	-1.07	0.28
**Δ Albumin%**	-13.53 (-19.98~-4.19)	-21.59 (-26.1~-15.23)	-2.33	**0.02**
**Postoperative complications**	12 (*n*=9)	19 (*n*=12)	0.003	0.61
Fever caused by infection	2	4		
Biliary leakage	1	0		
Hypoproteinemia	2	4		
Massive ascites	1	7		
Spontaneous bacterial peritonitis	1	1		
Hydrothorax	1	2		
Kaliopenia	3	0		
Pneumonia	0	1		
Incision seepage	1	0		

### Survival

**Fig. 3:**
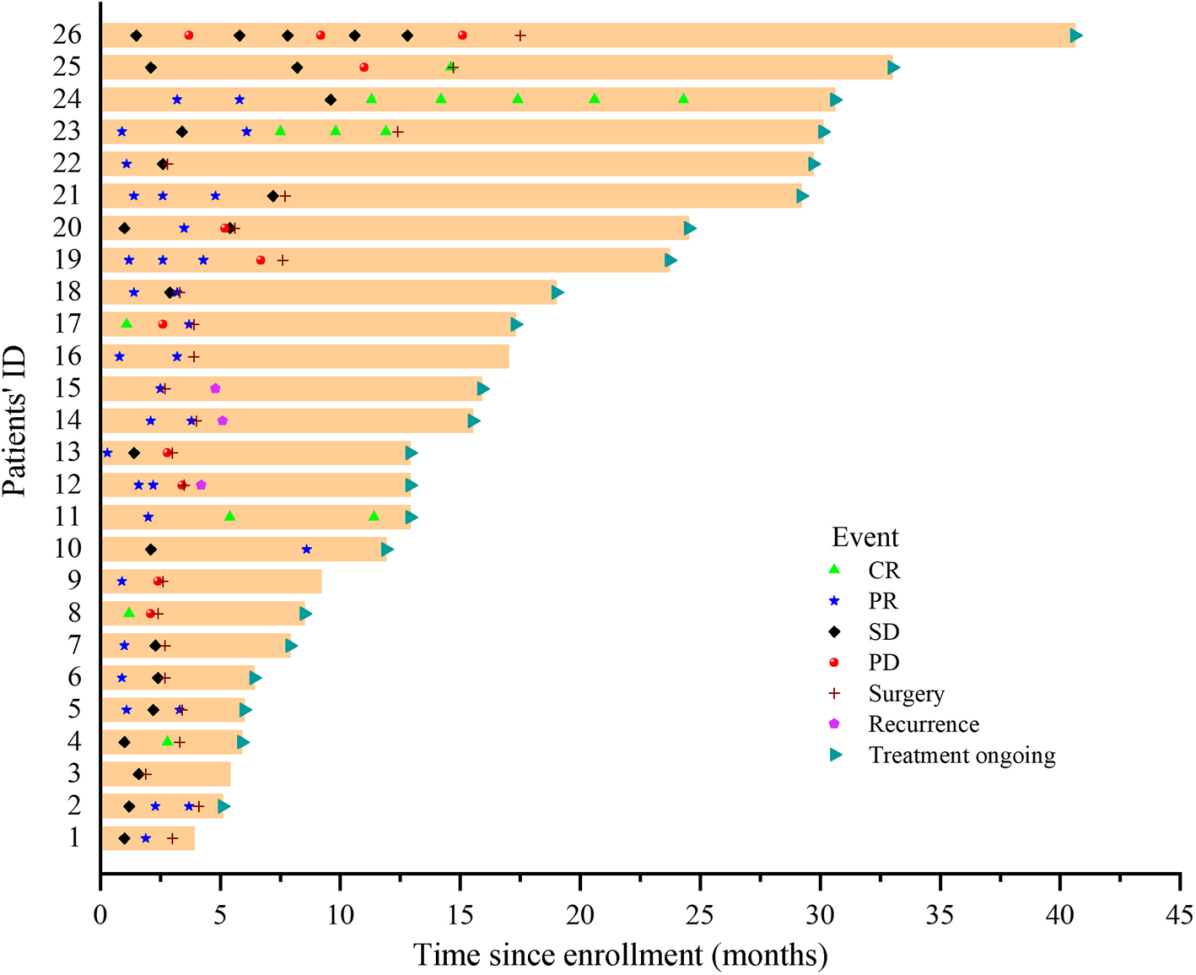
Survival of 26 patients. Information of tumor response at every evaluation (CR [complete response], PR [partial response], SD [stable disease] or PD [progressive disease]), surgery, recurrence after surgery and whether the adjuvant therapy was still ongoing were labelled with different markers

**Fig. 4:**
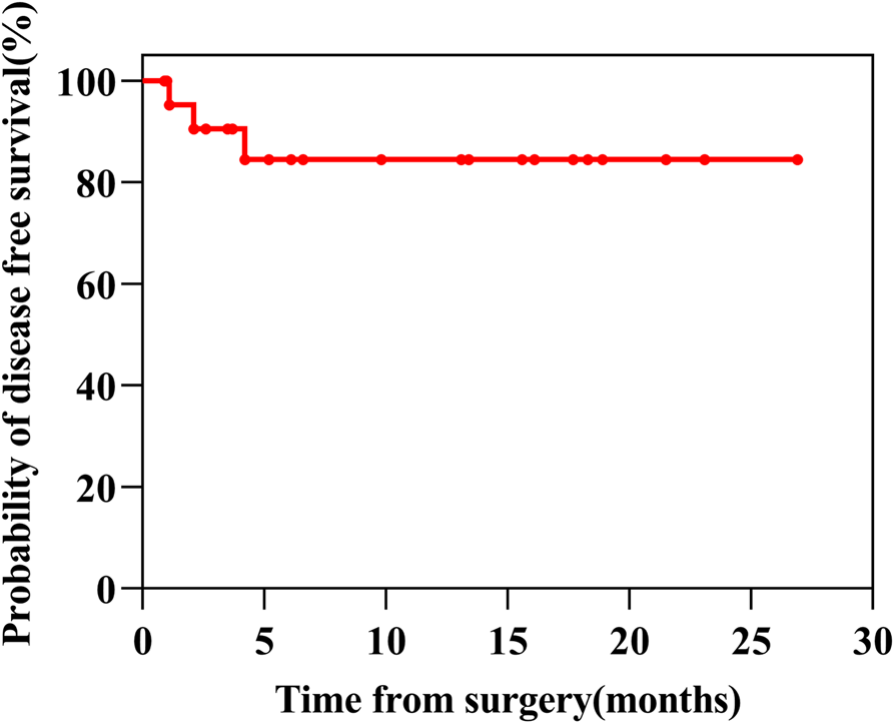
Kaplan-Meier analysis showing disease free survival (DFS) in successful resected uHCC patients initially

Survival information is presented in Figs. 3 and 4. At the latest follow-up, all 26 patients were alive, with the median follow-up reaching 12.9 months (range, 3.9~40.6) from diagnosis. For 23 patients who underwent conversion surgery, the median follow-up time was 11.4 months (range, 0.9~26.9) since surgery. 3 of 23 patients recurred after conversion surgery, with disease free survival (DFS) of 1.1, 2.1 and 4.2 months each. The remaining 20 patients are in DFS at their most recent follow-up. For the 3 patients who chose W&W, 2 remain in CR under mRECIST, with 1 initial PR patient ultimately experiencing PD. Nineteen of 23 patients continued to receive adjuvant treatment after the surgery, mainly (14/19) with only systemic treatment, and 5 also with TACE. The interval time (days after the surgery) before the initiation of the adjuvant therapy was 37.1±9.8 days. Information about the adjuvant therapy regimens is summarized in Table 8**.** The univariate and multivariate analyses of prognostic factors in 809 uHCC patients are illustrated in Table 9. Among 809 uHCC patients who underwent systemic and locoregional treatment, the univariate analysis showed that number of tumors, tumor size, liver cirrhosis and ascites were significantly associated with the successful conversion therapy. The multivariate Cox analysis showed that liver cirrhosis were significantly predictive factors for the successful conversion therapy of the patients (HR=0.291, 95%CI: 0.098~0.869, *P*=0.027).

**Table 8:** The specific therapeutical program of all patients before and after R0 resection

Patients	Conversion therapies	Adjuvant therapies	
		Modalities	Intervals (days after the surgery)
1	HAIC + Lenvatinib + Camrelizumab	No	-
2	HAIC + Lenvatinib + Camrelizumab	Lenvatinib + Camrelizumab	29
3	HAIC + Lenvatinib + Camrelizumab	No	-
4	HAIC + Lenvatinib + Camrelizumab	Lenvatinib + Tislelizumab	40
5	HAIC + Lenvatinib + Camrelizumab	Lenvatinib + Camrelizumab	33
6	HAIC + Lenvatinib + Camrelizumab	Lenvatinib + Camrelizumab	42
7	TACE + Lenvatinib + Camrelizumab	TACE+Lenvatinib + Tislelizumab	41
8	HAIC + Lenvatinib + Camrelizumab	Tislelizumab	43
9	HAIC + Lenvatinib + Camrelizumab	No	-
10W&W	HAIC + Apatinib + Camrelizumab	No	-
11W&W	HAIC + Apatinib + Camrelizumab	Camrelizumab	-
12	HAIC + Lenvatinib + Camrelizumab	TACE + Lenvatinib + Camrelizumab	33
13	HAIC + Lenvatinib + Camrelizumab	Lenvatinib + Camrelizumab	30
14	HAIC + Apatinib + Camrelizumab	TACE + Lenvatinib + Camrelizumab	41
15	TACE + Radiotherapy+ Lenvatinib	TACE + Lenvatinib	50
16	TACE + Lenvatinib + Camrelizumab	No	-
17	HAIC + Lenvatinib + Camrelizumab	Lenvatinib + Camrelizumab	35
18	TACE + Apatinib + Camrelizumab	Apatinib + Camrelizumab	37
19	TACE + Sorafenib + Camrelizumab	Camrelizumab	39
20	TACE + Apatinib + Camrelizumab	Apatinib + Camrelizumab	43
21	TACE + Lenvatinib	Lenvatinib	35
22	TACE + Lenvatinib	Lenvatinib	60
23	TACE + Sorafenib +Sintilimab	TACE + Sorafenib + Sintilimab	33
24W&W	TACE + Sorafenib + Camrelizumab	Sorafenib + Camrelizumab	-
25	Sorafenib+ Sintilimab	Sintilimab	28
26	TACE + Lenvatinib	Lenvatinib	12

*W&W* watch & wait

**Table 9:** Univariate and multivariate analyses model analyzed predictors of successful conversion in 809 patients.

**Variables**	**Univariate analysis**		**Multivariate analysis**	
	**HR (95%CI)**	***P* value**	**HR (95%CI)**	***P* value**
Age (years, < 65 *vs* ≥65)	0.629(0.213~1.86)	0.402		
Sex (female *vs* male)	0.95(0.281~3.212)	0.935		
ECOG PS (0~2 *vs* 3~4)	0.049(0.022~1.229)	0.769		
BCLC staging (A~B *vs* C~D)	1.134(0.435~2.953)	0.797		
Child-Pugh classification (A~B *vs* C)	0.384(0.051~2.883)	0.838		
Number of tumors (solitary *vs* multiple)	0.391(0.166~0.921)	**0.032**	0.738(0.278~1.956)	0.541
Tumor size (cm, ≤10 vs >10)	3.077(1.296~7.302)	**0.011**	2.484(0.892~6.92)	0.082
AFP(ng/ml,≤400 *vs* >400)	2.122(0.796~5.653)	**0.133**	2.099(0.766~5.751)	0.15
HBV (no *vs* yes)	0.451(0.16~1.268)	**0.131**	0.63(0.218~1.815)	0.63
ALT (u/L,≤40 *vs* >40)	0.704(0.264~1.876)	0.483		
AST(u/L, ≤35 *vs* >35)	2.375(0.687~8.204)	**0.172**	2.132(0.575~7.905)	0.257
Bilirubin(umol/L, ≤35 *vs* >35)	0.042(0~29.086)	0.342		
Albumin (g/L, ≤35 *vs* >35)	0.94(0.272~3.248)	0.922		
Liver cirrhosis (no *vs* yes)	0.165(0.06~0.454)	<**0.001**	0.291(0.098~0.869)	**0.027**
PVTT (no *vs* yes)	1.18(0.489~2.85)	0.713		
HVTT (no *vs* yes)	2.501(0.58~10.782)	0.219		
IVCTT (no *vs* yes)	0.045(0~93.26)	0.425		
Ascites (no *vs* yes)	0.229(0.053~0.987)	**0.048**	0.259(0.056~1.206)	0.085
Intrahepatic metastasis (no *vs* yes)	0.301(0.04~2.25)	0.242		
Extrahepatic metastasis (no *vs* yes)	0.148(0.054~0.993)	**0.063**	0.181(0.024~1.378)	0.099
lymphatic metastasis (no *vs* yes)	0.672(0.252~2.012)	0.478		
Treatment (LR+TKI/anti-PD-1 *vs* LR+TKI+anti-PD-1)	1.71(0.584~5.002)	0.328		

The association between successful conversion and those baseline stratification tactors, including age, sex, ECOG, BCLC staging, Child-Pugh classification, number of tumors, tumor size, serum AFP level, HBV infection, ALT, AST, bilirubin, albumin, liver cirrhosis, PVTT, HVTT, IVCTT, ascites, Intrahepatic metastasis, lymphatic metastasis, extrahepatic metastases and treatment methods, respectively, was analyzed by Cox proportional-hazards model to generate crude HRs and 95% Cls. Variables with *P* < 0.2 in univariate analysis were included in multivariate regression analysis model. *P* < 0.05 was considered to indicate a statistically significant

*BCLC* Barcelona Clinic Liver Cancer Staging System, *PVTT* Portal vein tumor thrombus, *HVTT* Hepatic vein tumor thrombus, *IVCTT* Inferior vena cava tumor thrombus, *LR* Loco-regional therapy

## Discussion

In the global HCC BRIDGE study inclusive of HCC patients diagnosed between 2005 and 2011 from 20 sites worldwide, the percentage of patients “ideal for resection” was approximately 10% [16]. Among all 1,904 HCC patients admitted to our center in the recent 3 years, 328 patients (17%) were R0-resectable. This higher local proportion of operable patients may result from improved screening practices in a high-risk population, health examination and national healthcare policy, but it also could because we are a surgical department. And yes, the vast majority of patients identified in this study remain uHCC; for them, the direct curative-intended resection was not possible, thus the conversion therapy worth more attention.

Conversion surgery in HCC is not a novel concept. Tang et al. reported that over half century ago, 12.8% uHCC patients were “down-staged and resected”, and their 5-year survival rate reached 48.7% [17]. Their conversion therapies included hepatic artery ligation by the open surgery followed by chemotherapeutic infusion, radiotherapy and radioimmunotherapy or various combinations therein. The progress in the systemic treatment of uHCC brings novel and promising choices to the therapeutic modalities of conversion therapy [18].

In the recent clinical trials and the cohort studies, the conversion rate varied from 15.9% [8] to as high as 60% [19] with this variation likely related to the variability in inclusion criteria. In clinical trials of conversion surgery, the conversion rates are normally set as one of the endpoints, the recruited patients received treatment with the definite aim of being converted to resectable HCC. While in this study, we provide data on the rates of conversion resectability in all-comers.

In clinical practice, when the treatment of a uHCC starts, whether the aim is to convert or just to prolong the OS cannot always be set clearly; instead, it can be a policy of “treat and see”; if a patient responses well to the treatment and the curative resection becomes possible, he will undergo resection; otherwise the current treatment continues, or changes to the subsequent-line therapy. So, it is difficult to define the “conversion-intended” uHCC population, which is the denominator when calculating the conversion rate. So, the conversion rates from clinical trials cannot indicate the conversion rate accurately and objectively in the real-world clinical practice.

In our real-world data, we did not calculate the conversion rate upon “conversion-intended” patients. The conversion rate from all 1344 treated uHCC patients was 1.93%. The conversion rates in our real-world practice may not be as high as expected, but considering that only 10% to 20 % HCC patients are ideally resectable when diagnosed, a conversion rate of about 2% in uHCC patients is still a substantial progress. For uHCC patients who received intensive treatment, defined as systemic plus loco-regional treatment, the conversion rate increased to 3.09%. When systemic therapies were combined with loco-regional treatment, tumor responses increase significantly; on the other hand, clinicians are more likely to prescribe intensive treatment to patients judged to have an increased likelihood of being converted to resection, eg. better performance score and liver reservation, non-widely spreading tumors, etc.

During the conversion therapy, AEs of any grade occurred in nearly all patients, half patients suffered TRAEs over grade 3. Abnormal liver functions were often observed after loco-regional treatment (esp. HAIC). Although the incidence of ≥grade 3 TRAEs was relatively high, the symptoms were alleviated by symptomatic treatment and/or dose adjustment in all patients. Surgeries after conversion therapy also showed satisfactory safety; conversion therapies did not increase the mortality and morbidity incidence. Extra operative difficulties and trauma were probably the results of more advanced tumor stages of the conversion group.

The general tumor response in 26 patients were very good. Twenty-five out of 26 patients showed decrease in the size of the target lesion, ORR was 42.3% or 76.9% under RECIST v.1.1 or mRECIST criteria, respectively. Notably, the final pCR confirmed by the post-operative pathologic examination was not in exact accordance with the RECIST or mRECIST results; by mRECIST, 2 out of 6 SD, 4 out of 15 PR and 3 out of 5 CR patients reached pCR. To better assess the tumor response of HCC by clinical parameters, we have suggested that the criteria for determining “clinical complete response (cCR)” more accurately in HCC should be proposed [20].

Among 5 patients achieved CR (mRECIST) at the final evaluation, 3 CR patients underwent R0 resection and remained in DFS at the latest follow-up. The other 2 CR patients chose the “watch & wait” (WW) strategy, and have been in tumor-free status for 7.6 and 19.7 months, respectively.

In patients with rectal and esophageal cancer, when neoadjuvant therapy results in clinical complete response (cCR), the questions remain as to whether surgery can be avoided. In the latest NCCN guideline, a “watch & wait” (WW) strategy is also an optional choice for rectal cancer patients with cCR. Now the same question is asked in HCC patients in the conversion therapy, and this question has strong clinical implications [20]. For HCC patients who reach cCR during the conversion therapy, for those with borderline liver functional reserve, or relatively large tumor that need major hepatectomy, or have other high risks of postoperative mortality and morbidities, instead of surgical resection, WW policy can be another choice that worth being considered.

Patients with BCLC stage C with EHM (CNLC stage IIIb), are not typically considered for conversion therapy [7], because the EHM lesion often indicates extensive dissemination, such that even extra-hepatic oligo-metastasis may not be resectable removed during the index hepatectomy. In this study, all 5 converted patients with oligo-EHM remained in DFS at their final follow-up. In the criteria for cCR of HCC we proposed, we’ve also considered the evaluation of EHM [20]. Instead of parameters from tumors, the only positive variable in the multivariate regression is the presence of liver cirrhosis. Better liver function permits more intensive anti-HCC treatment, then brings more chance for the conversion.

This study has several limitations. First, is its retrospective nature within a single surgical oncologic liver centre. Although the scales of the study were large, but only a small number of patients finally reached the conversion resection, so the findings may not be generally representative. Secondly, the length of follow-up is short, so the survival data of these patients are not available yet and definitive cure rate cannot be accurately understood. Finally, this type of clinical study cannot confirm the rationale of conversion surgery. Convertible uHCC patients are typically a minority sub-group that respond well to the anti-HCC therapies, so the conversion therapy selects tumors with favourable biology such that it is difficult to understand whether patients have ultimately benefited from surgery. Nonetheless, given the small proportion of patients who reach conversion surgery, analysis in a randomized trial setting is unlikely to be feasible in the foreseeable future due to the large sample size that would need to be required.

## Supplementary Information

**Additional file 1. **

## Data Availability

The datasets used and/or analysed during the current study available from the corresponding author on reasonable request.

## References

[1] Rumgay H, Arnold M, Ferlay J, Lesi O, Cabasag CJ, Vignat J, Laversanne M, McGlynn KA, Soerjomataram I (2022). Global burden of primary liver cancer in 2020 and predictions to 2040. J Hepatol.

[2] Sung H, Ferlay J, Siegel RL, Laversanne M, Soerjomataram I, Jemal A, Bray F (2021). Global Cancer Statistics 2020: GLOBOCAN Estimates of Incidence and Mortality Worldwide for 36 Cancers in 185 Countries. CA Cancer J Clin.

[3] Cheng AL, Qin S, Ikeda M, Galle PR, Ducreux M, Kim TY, Lim HY, Kudo M, Breder V, Merle P (2022). Updated efficacy and safety data from IMbrave150: Atezolizumab plus bevacizumab vs. sorafenib for unresectable hepatocellular carcinoma. J Hepatol.

[4] Finn R, Kudo M, Merle P, Meyer T, Qin S, Ikeda M, Xu R, Edeline J, Ryoo B, Ren Z (2022). LBA34 Primary results from the phase III LEAP-002 study: Lenvatinib plus pembrolizumab versus lenvatinib as first-line (1L) therapy for advanced hepatocellular carcinoma (aHCC). Annals of Oncology.

[5] Finn RS, Qin S, Ikeda M, Galle PR, Ducreux M, Kim TY, Kudo M, Breder V, Merle P, Kaseb AO (2020). Atezolizumab plus Bevacizumab in Unresectable Hepatocellular Carcinoma. N Engl J Med.

[6] Villanueva A (2019). Hepatocellular Carcinoma. N Engl J Med.

[7] Sun HC, Zhou J, Wang Z, Liu X, Xie Q, Jia W, Zhao M, Bi X, Li G, Bai X (2022). Chinese expert consensus on conversion therapy for hepatocellular carcinoma (2021 edition). Hepatobiliary Surg Nutr.

[8] Zhu XD, Huang C, Shen YH, Ji Y, Ge NL, Qu XD, Chen L, Shi WK, Li ML, Zhu JJ (2021). Downstaging and Resection of Initially Unresectable Hepatocellular Carcinoma with Tyrosine Kinase Inhibitor and Anti-PD-1 Antibody Combinations. Liver Cancer.

[9] Zhu XD, Huang C, Shen YH, et al. Hepatectomy After Conversion Therapy Using Tyrosine Kinase Inhibitors Plus Anti-PD-1 Antibody Therapy for Patients with Unresectable Hepatocellular Carcinoma. Ann Surg Oncol. 2023;30(5):2782–90. 10.1245/s10434-022-12530-z PMC10085942 36178565

[10] Zhang W, Hu B, Han J, Wang Z, Ma G, Ye H, Yuan J, Cao J, Zhang Z, Shi J (2021). Surgery After Conversion Therapy With PD-1 Inhibitors Plus Tyrosine Kinase Inhibitors Are Effective and Safe for Advanced Hepatocellular Carcinoma: A Pilot Study of Ten Patients. Front Oncol.

[11] Qu WF, Ding ZB, Qu XD, et al. Conversion therapy for initially unresectable hepatocellular carcinoma using a combination of toripalimab, lenvatinib plus TACE: real-world study. BJS Open. 2022;6(5):zrac114. 10.1093/bjsopen/zrac114 PMC9499852 36125345

[12] Luo L, Xiao Y, Zhu G, Huang A, Song S, Wang T, Ge X, Xie J, Deng W, Hu Z (2022). Hepatic arterial infusion chemotherapy combined with PD-1 inhibitors and tyrosine kinase inhibitors for unresectable hepatocellular carcinoma: A tertiary medical center experience. Front Oncol.

[13] Marrero JA, Kulik LM, Sirlin CB, Zhu AX, Finn RS, Abecassis MM, Roberts LR, Heimbach JK (2018). Diagnosis, Staging, and Management of Hepatocellular Carcinoma: 2018 Practice Guidance by the American Association for the Study of Liver Diseases. Hepatology.

[14] Department of Medical Administration NH (2020). Health Commission of the People's Republic of C: [Guidelines for diagnosis and treatment of primary liver cancer in China (2019 edition)]. Zhonghua Gan Zang Bing Za Zhi.

[15] He M, Li Q, Zou R, Shen J, Fang W, Tan G, Zhou Y, Wu X, Xu L, Wei W (2019). Sorafenib Plus Hepatic Arterial Infusion of Oxaliplatin, Fluorouracil, and Leucovorin vs Sorafenib Alone for Hepatocellular Carcinoma With Portal Vein Invasion: A Randomized Clinical Trial. JAMA Oncol.

[16] Roayaie S, Jibara G, Tabrizian P, Park JW, Yang J, Yan L, Schwartz M, Han G, Izzo F, Chen M (2015). The role of hepatic resection in the treatment of hepatocellular cancer. Hepatology.

[17] Tang ZY, Zhou XD, Ma ZC, Wu ZQ, Fan J, Qin LX, Yu Y (2004). Downstaging followed by resection plays a role in improving prognosis of unresectable hepatocellular carcinoma. Hepatobiliary Pancreat Dis Int.

[18] Zhao L, Zhao H (2020). Conversion surgery for hepatocellular carcinoma in the new era of targeted and immune checkpoint inhibitor therapies. Hepatobiliary Surg Nutr.

[19] Zhang J, Zhang X, Mu H, Yu G, Xing W, Wang L, Zhang T (2021). Surgical Conversion for Initially Unresectable Locally Advanced Hepatocellular Carcinoma Using a Triple Combination of Angiogenesis Inhibitors, Anti-PD-1 Antibodies, and Hepatic Arterial Infusion Chemotherapy: A Retrospective Study. Front Oncol.

[20] Zhao L, Zhao H, Sun H (2022). It's time to propose a uniform criteria for determining "clinical complete response" in hepatocellular carcinoma. Hepatobiliary Surg Nutr.

