# Supplementary material for: Real-world practice of conversion surgery for unresectable hepatocellular carcinoma - a single center data of 26 consecutive patients
Source: BMC Cancer. 2023 May 20;23:465. doi: 10.1186/s12885-023-10955-7 (PMC10199571; doi:10.1186/s12885-023-10955-7)
Supplement: Supplementary file 1 — Additional file 1. [file 12885_2023_10955_MOESM1_ESM.pdf]

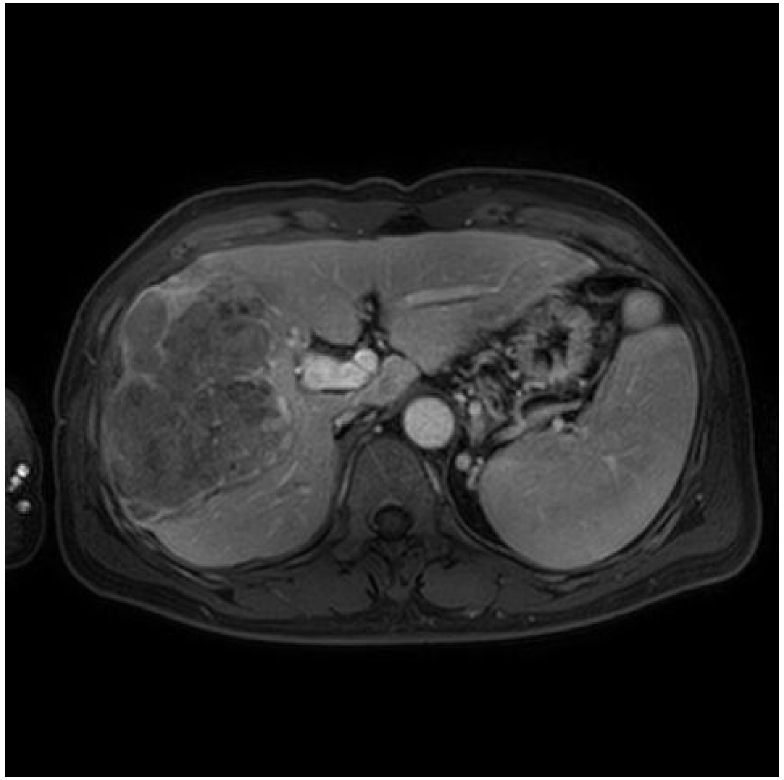

Before conversion

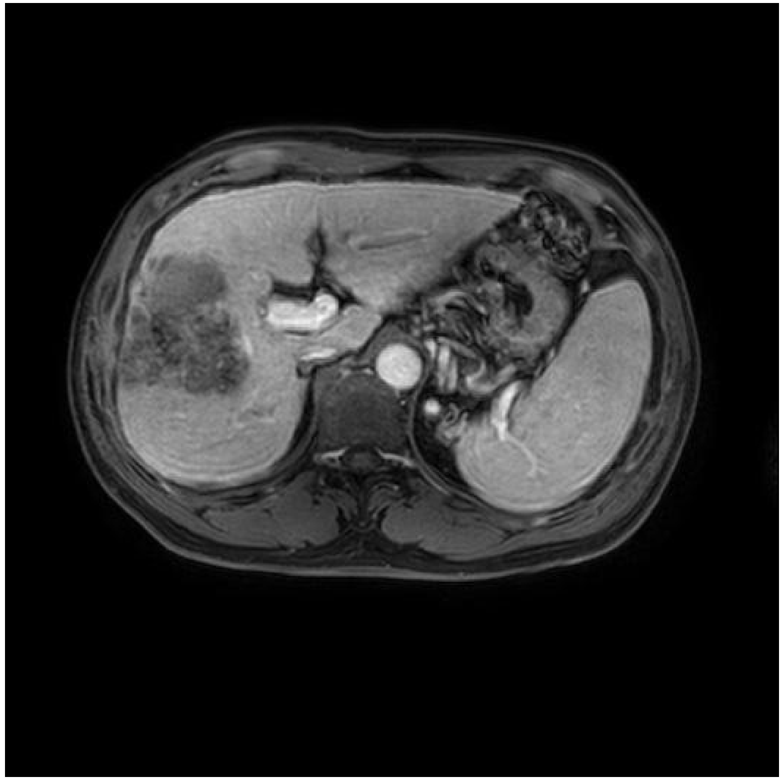

After conversion

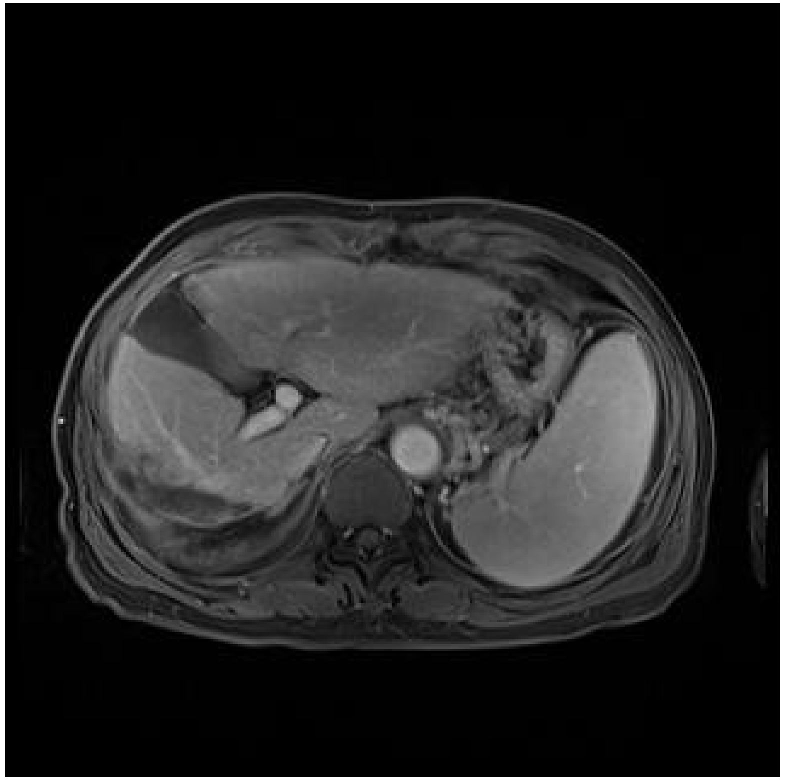

After surgery

Patient 01

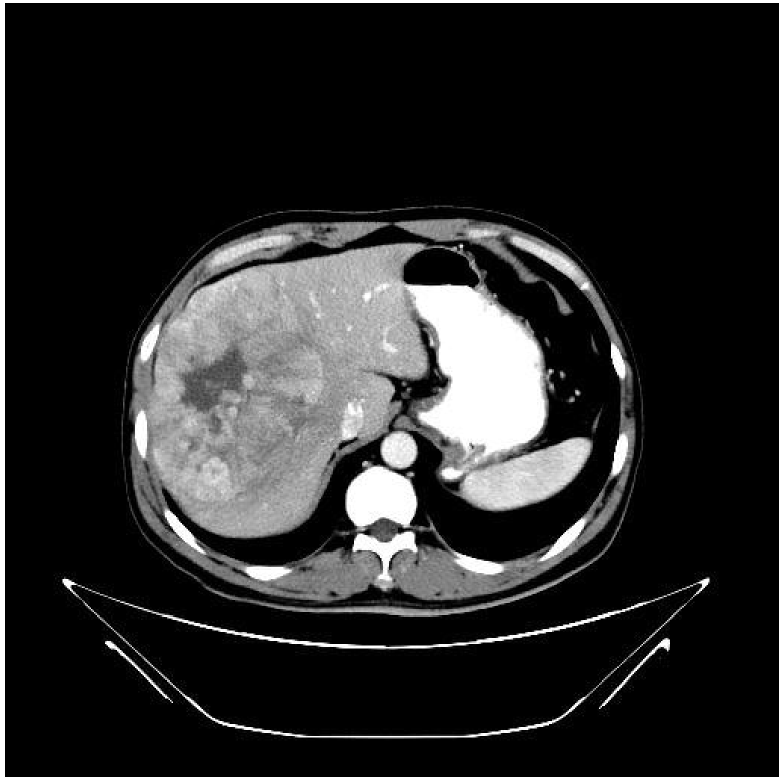

Before conversion

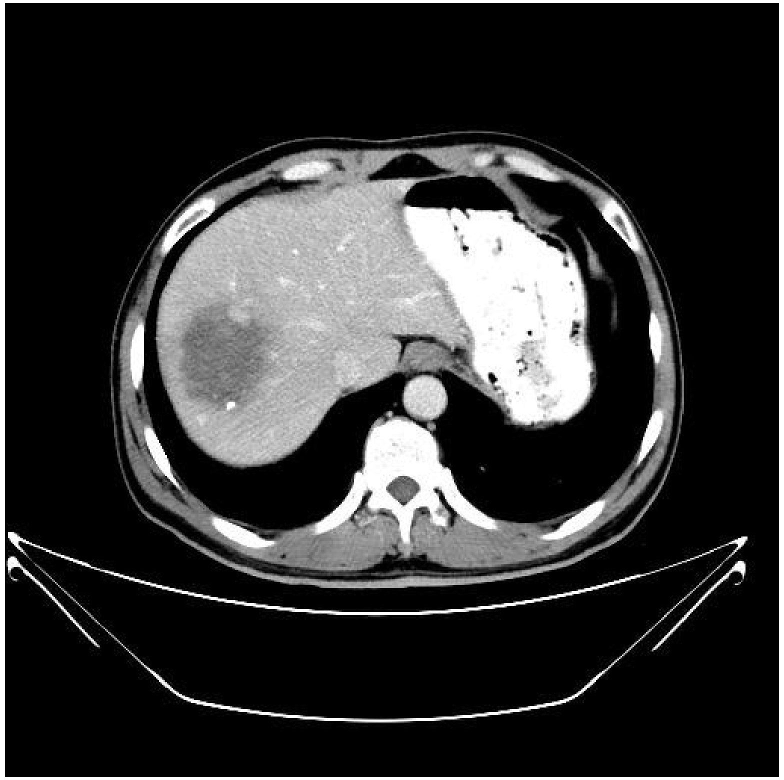

After conversion

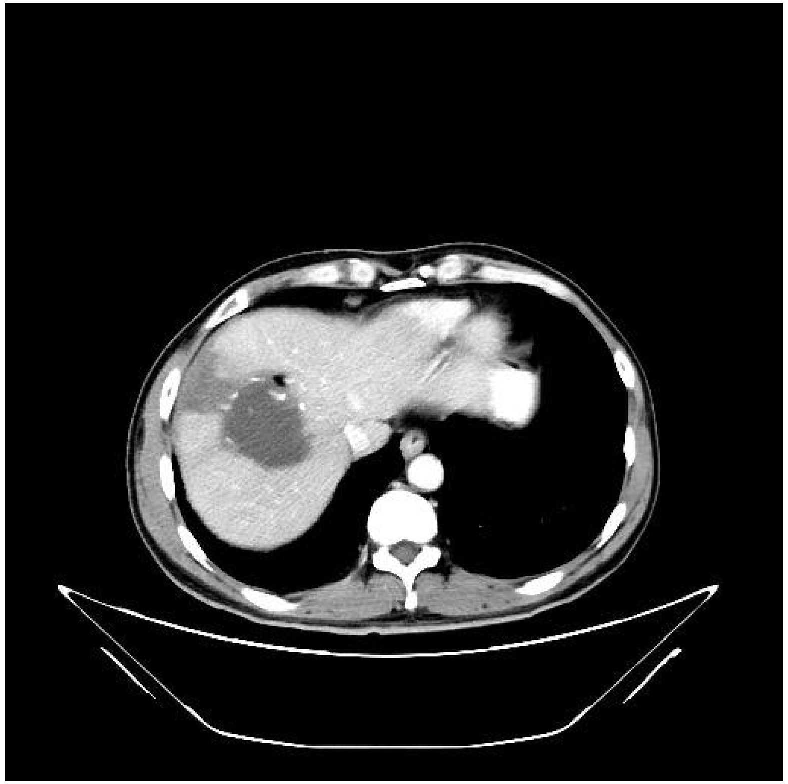

After surgery

Patient 02

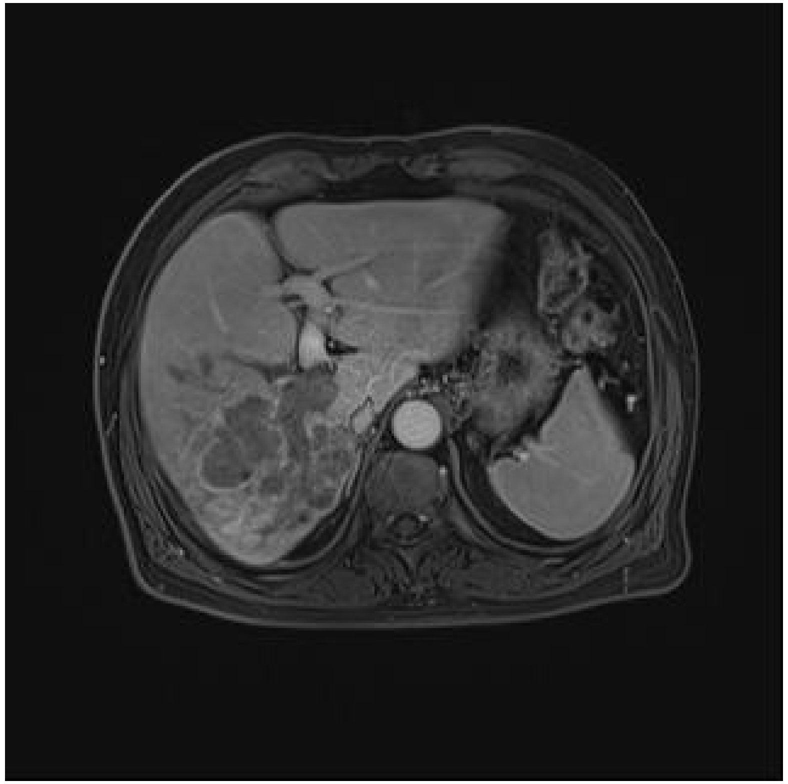

Before conversion

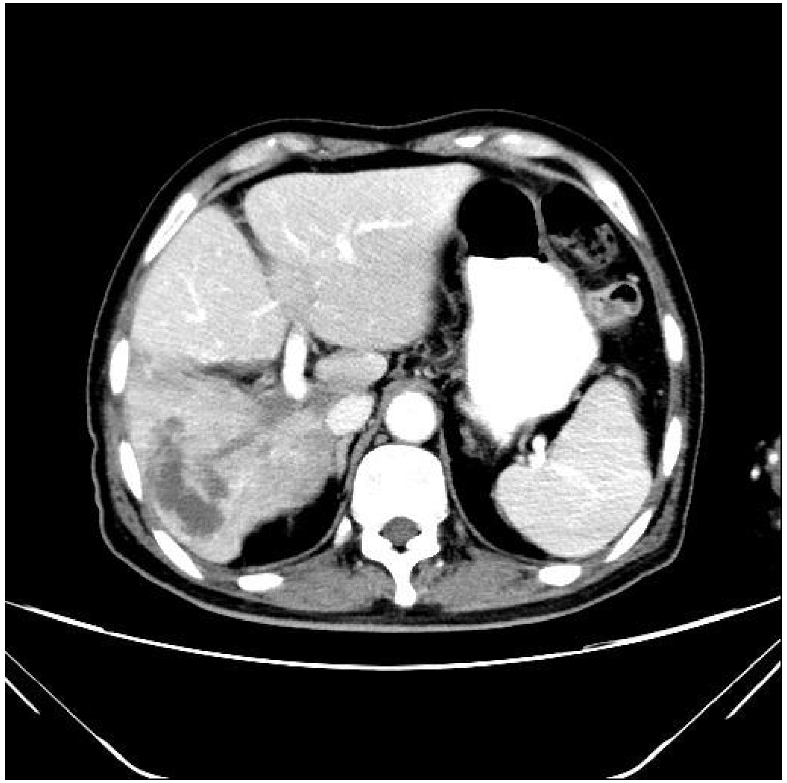

After conversion

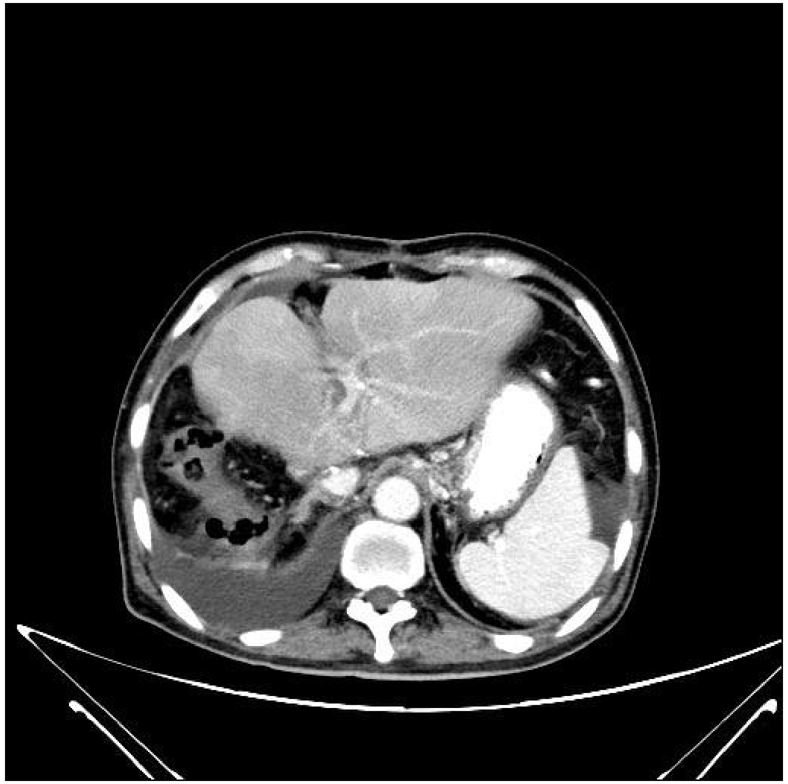

After surgery

Patient 03

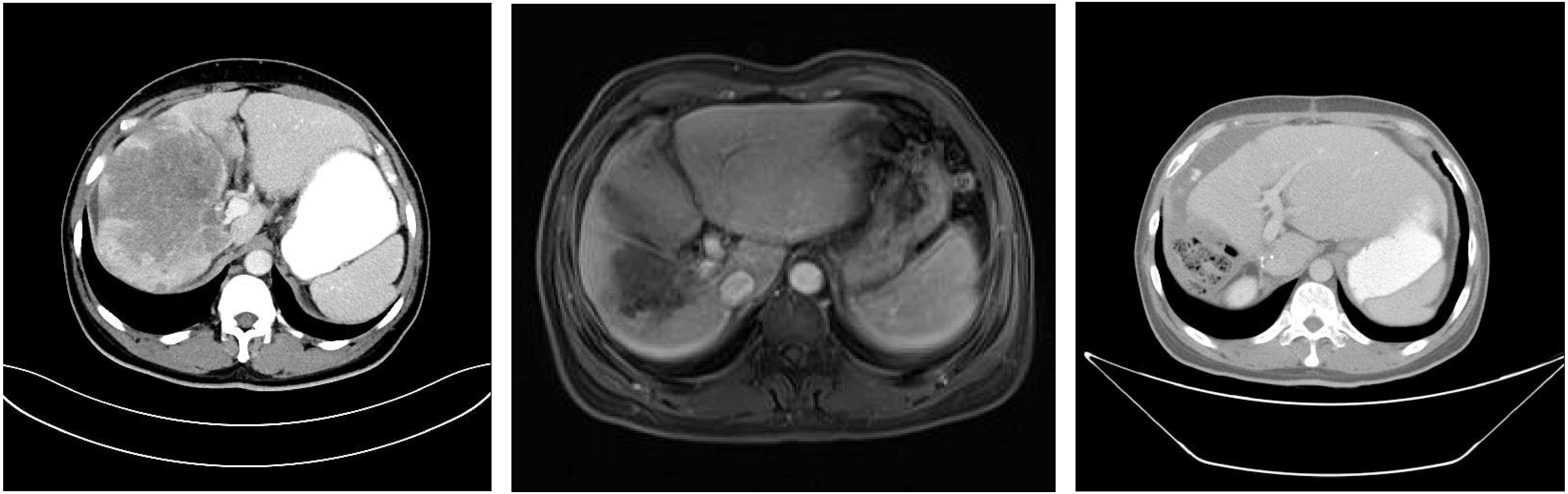

Before conversion After conversion After surgery

Patient 04

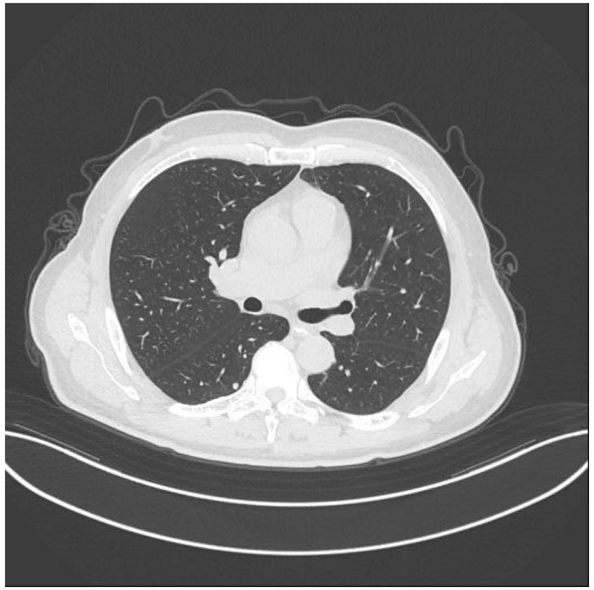

Before conversion

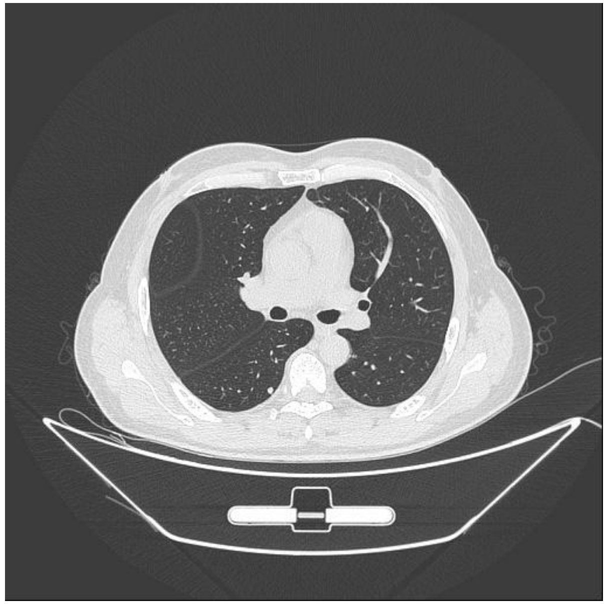

After conversion

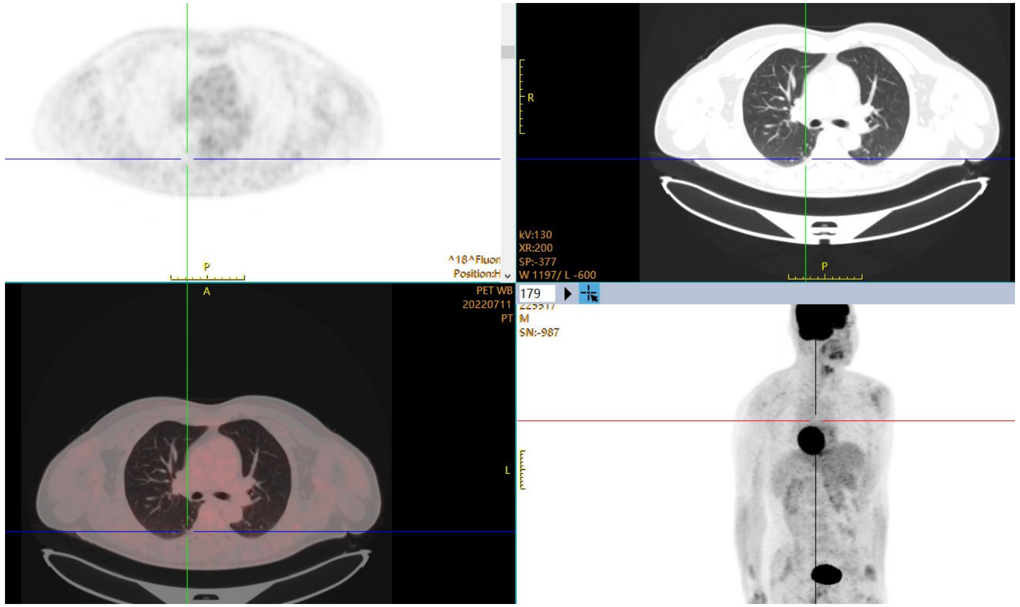

After surgery

Patient 04

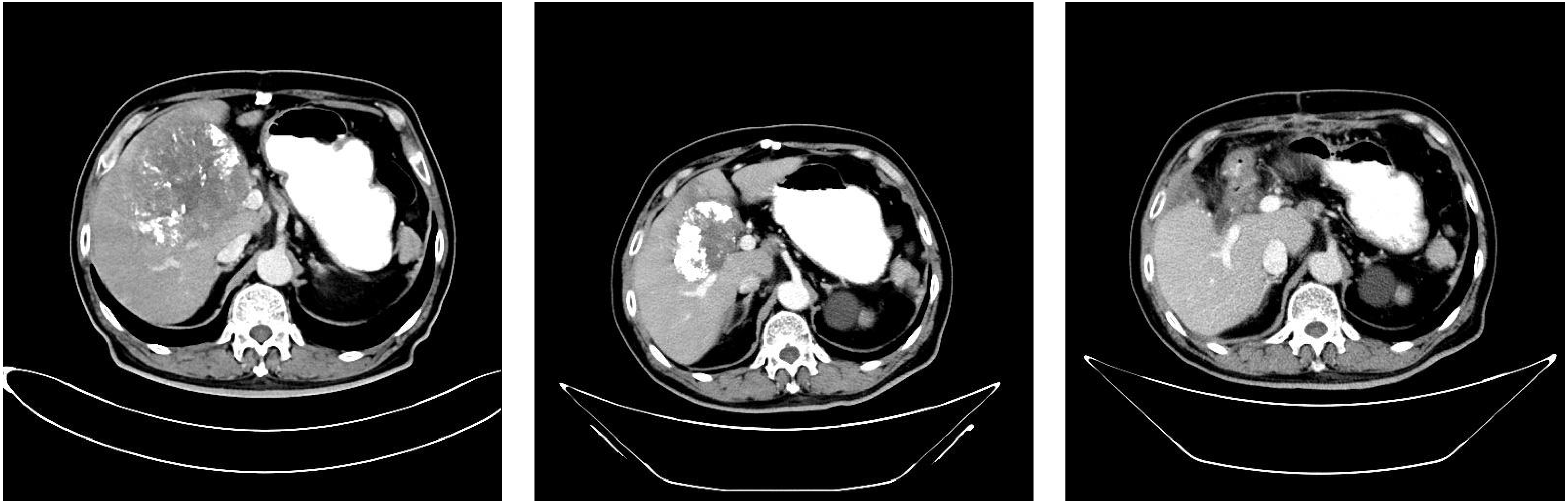

Before conversion

After conversion

After surgery

Patient 05

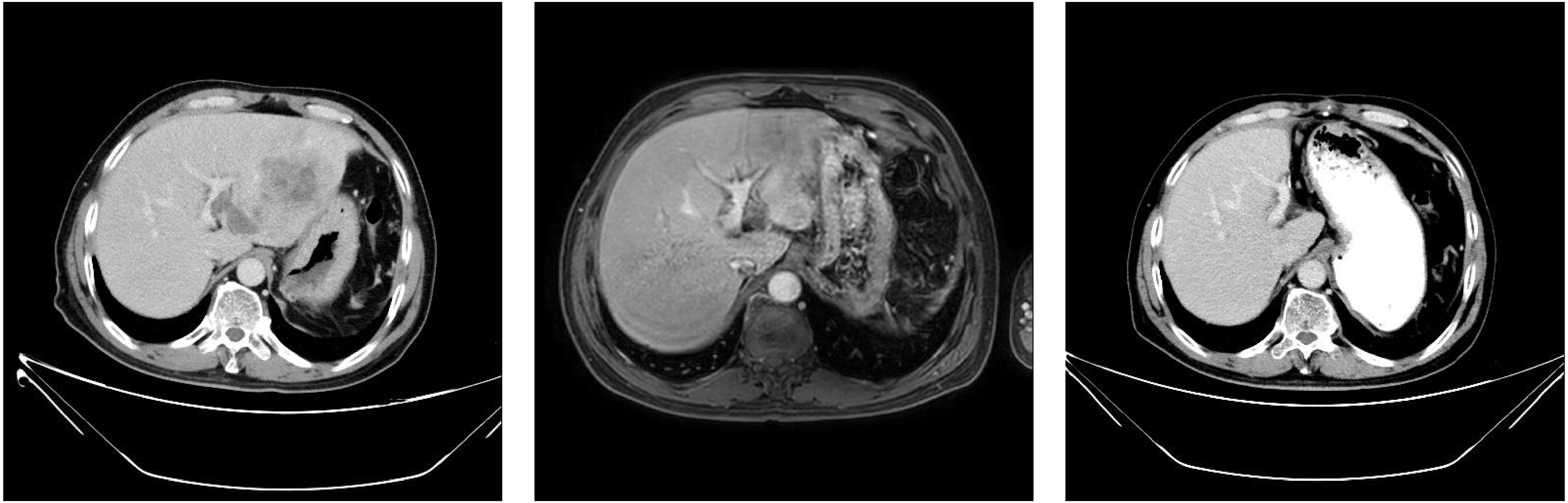

Before conversion | After conversion | After surgery

Patient 06

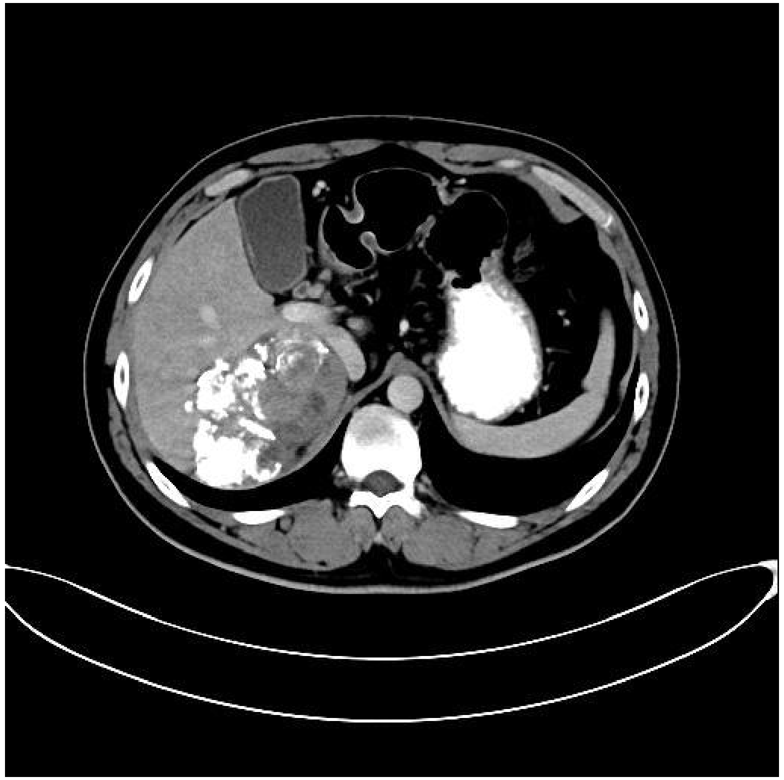

Before conversion

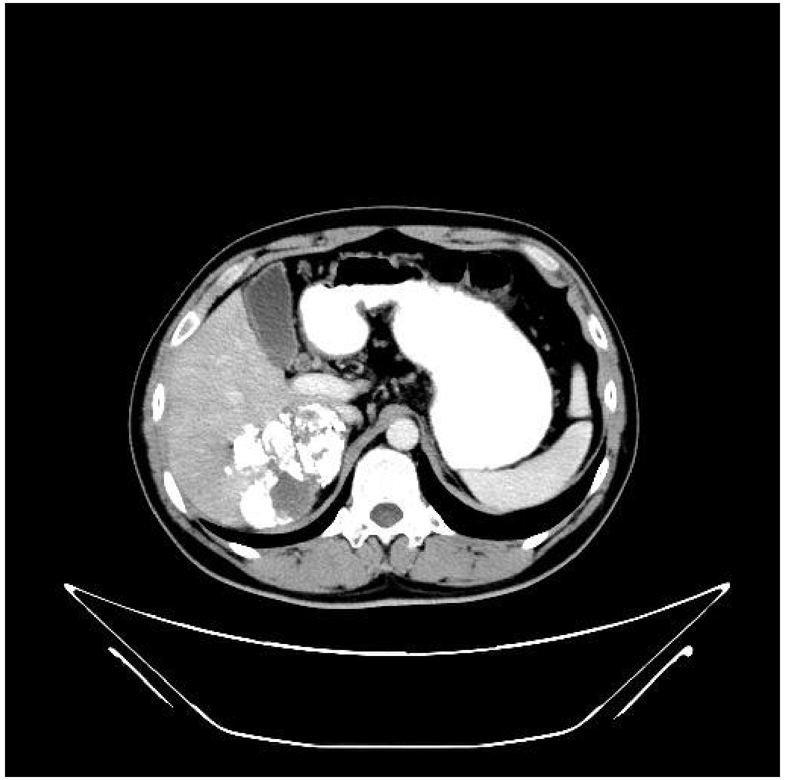

After conversion

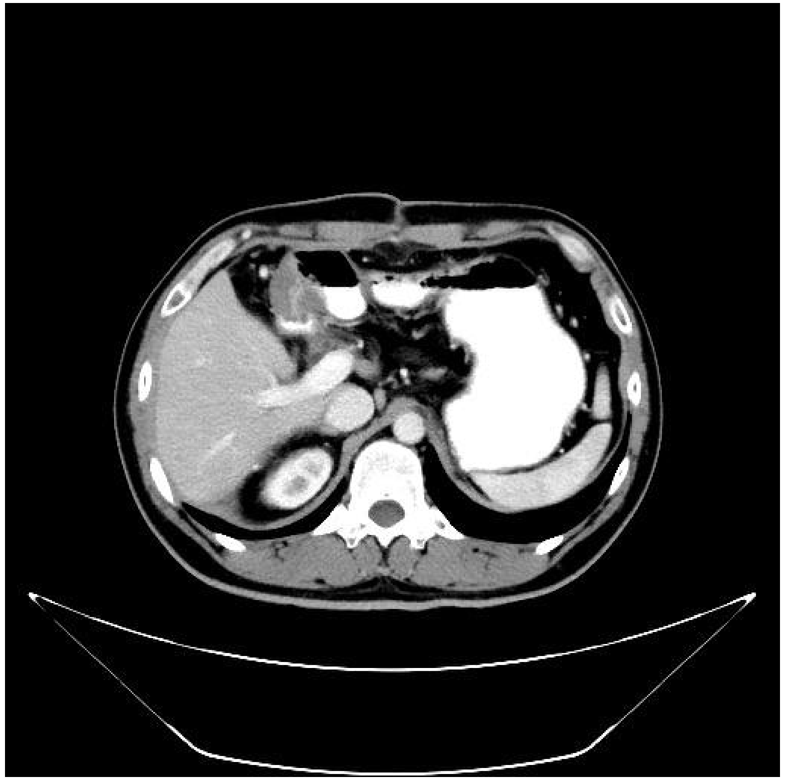

After surgery

Patient 07

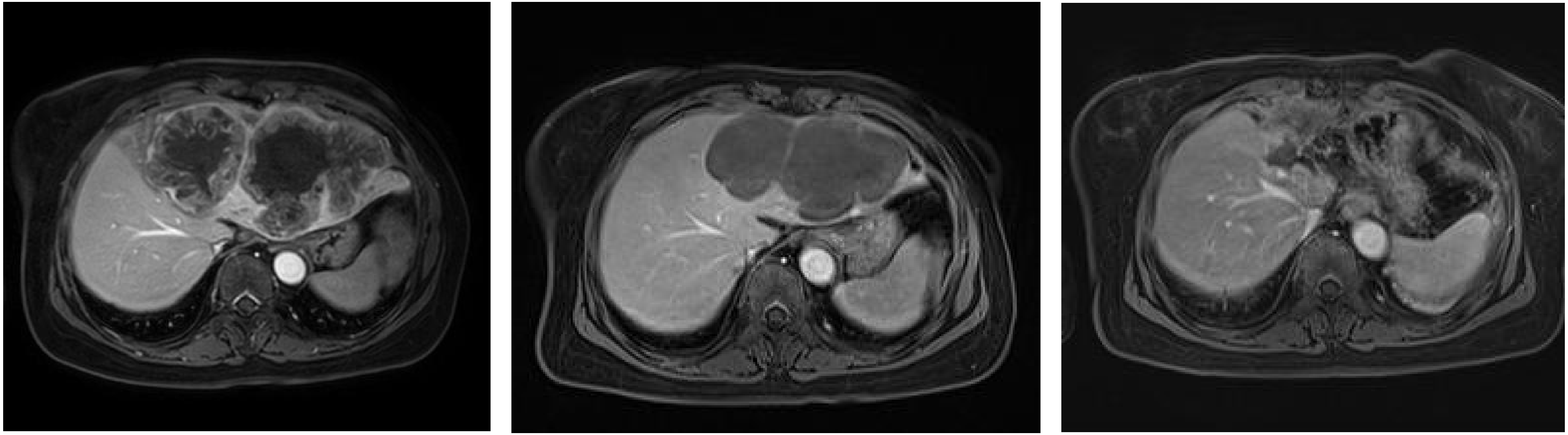

Before conversion After conversion After surgery

Patient 08

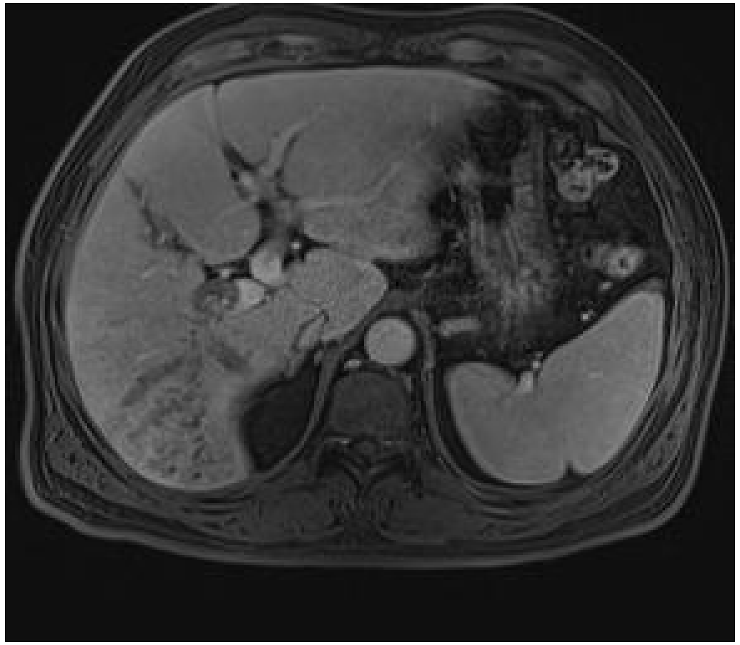

Before conversion

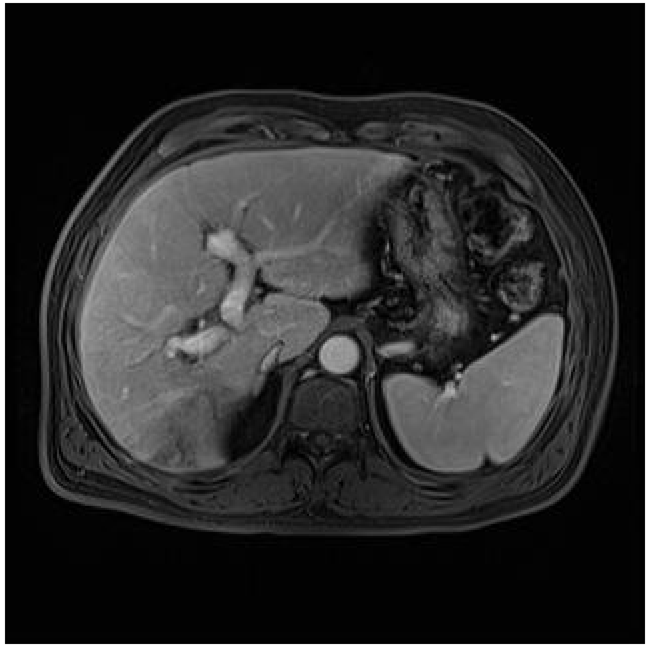

After conversion

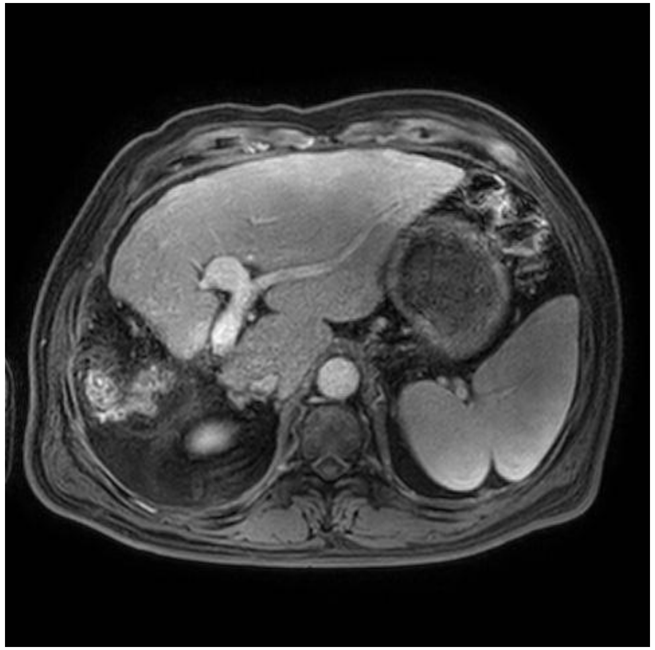

After surgery

Patient 09

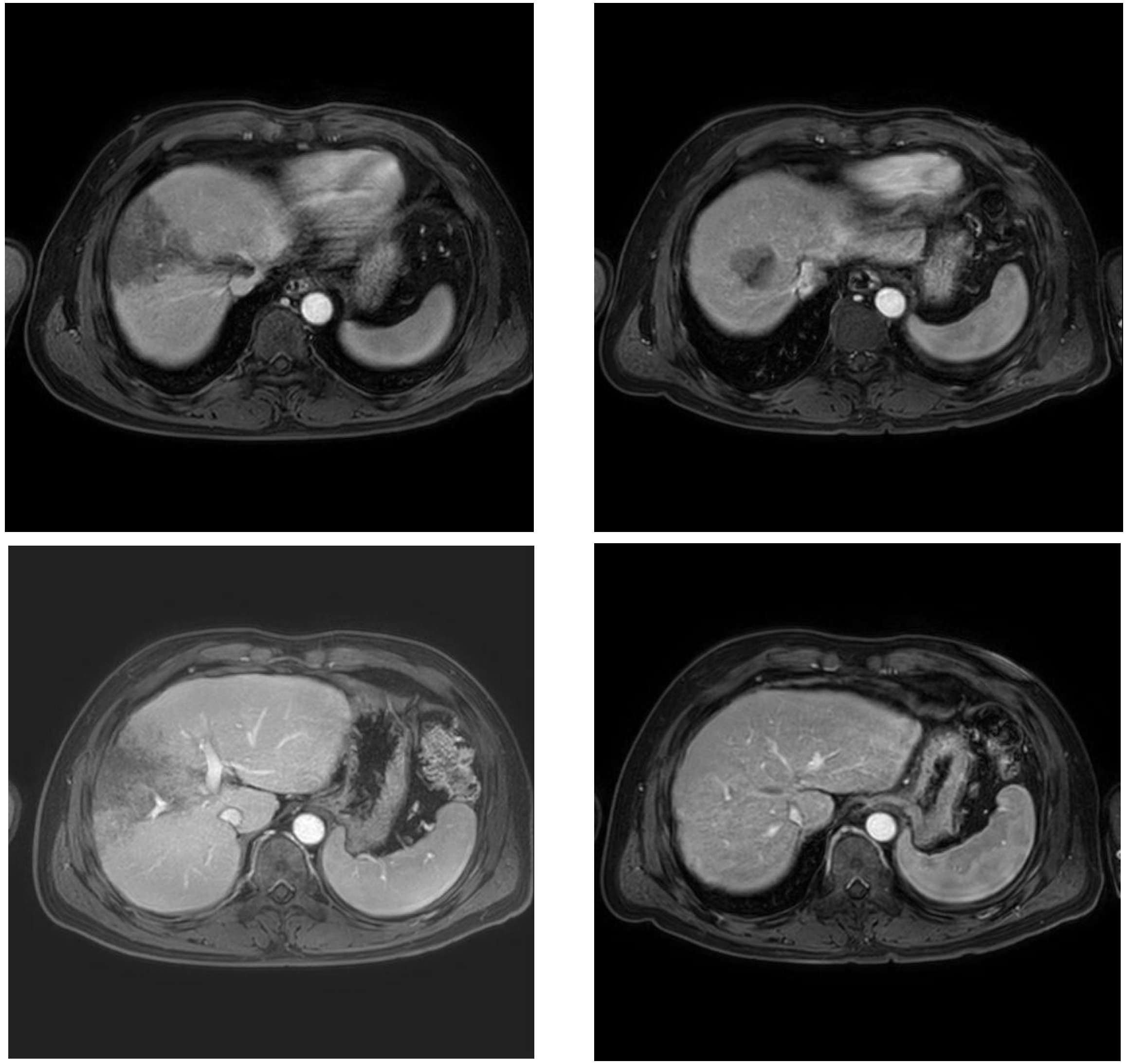

Before conversion After conversion

Patient 10

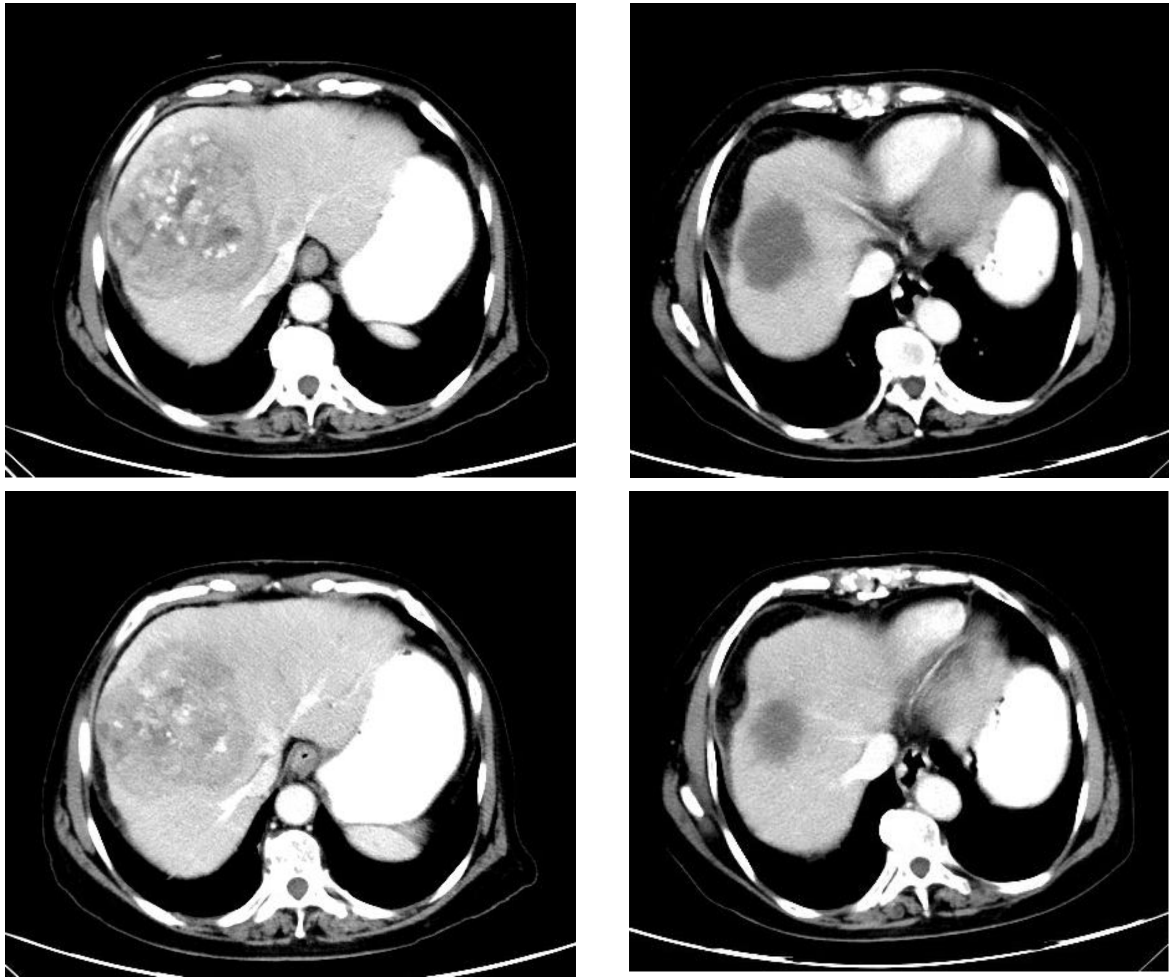

Before conversion After conversion

Patient 11

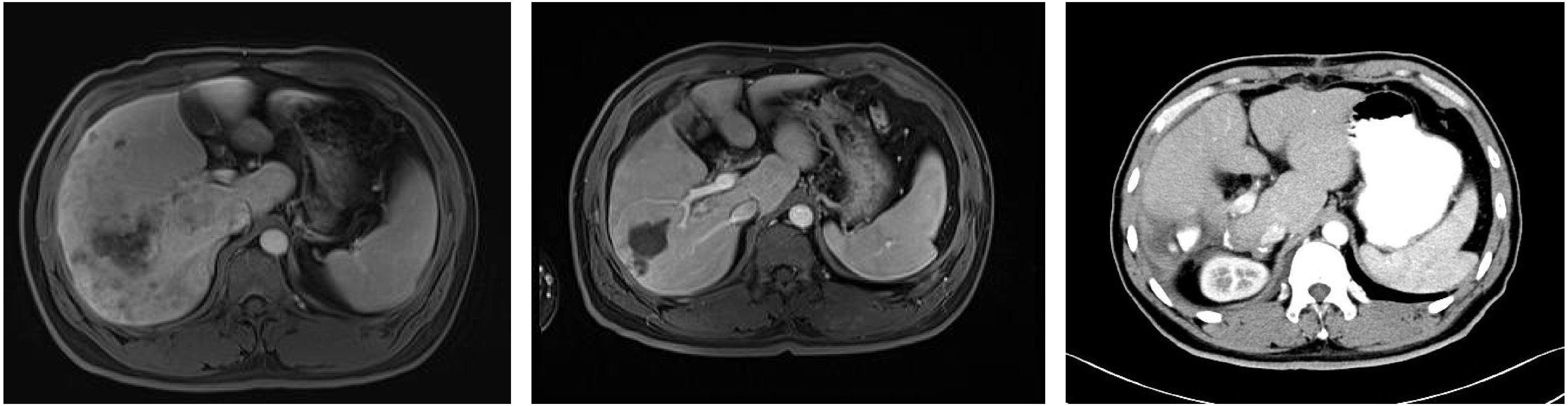

Before conversion After conversion After surgery

Patient 12

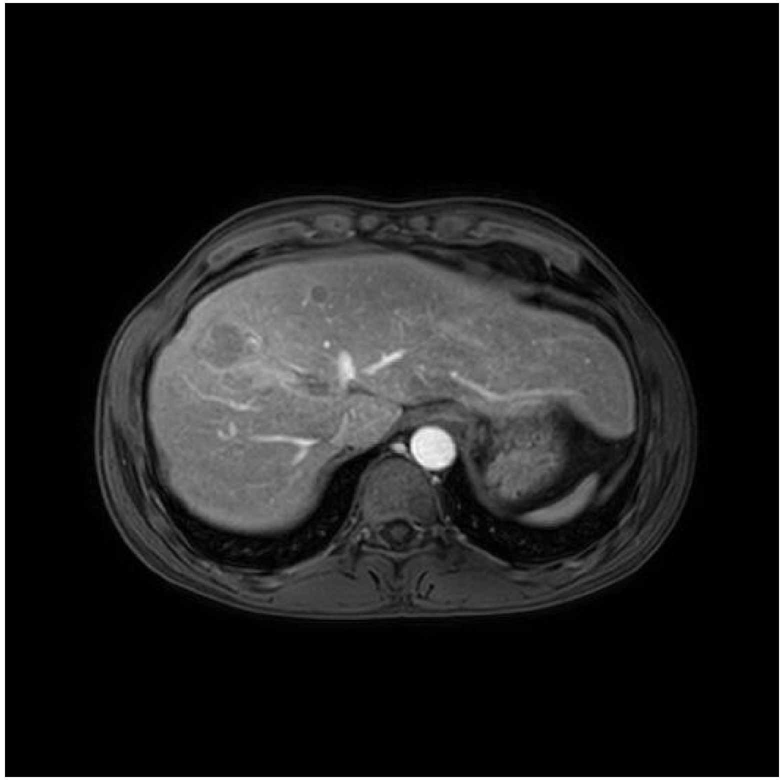

Before conversion

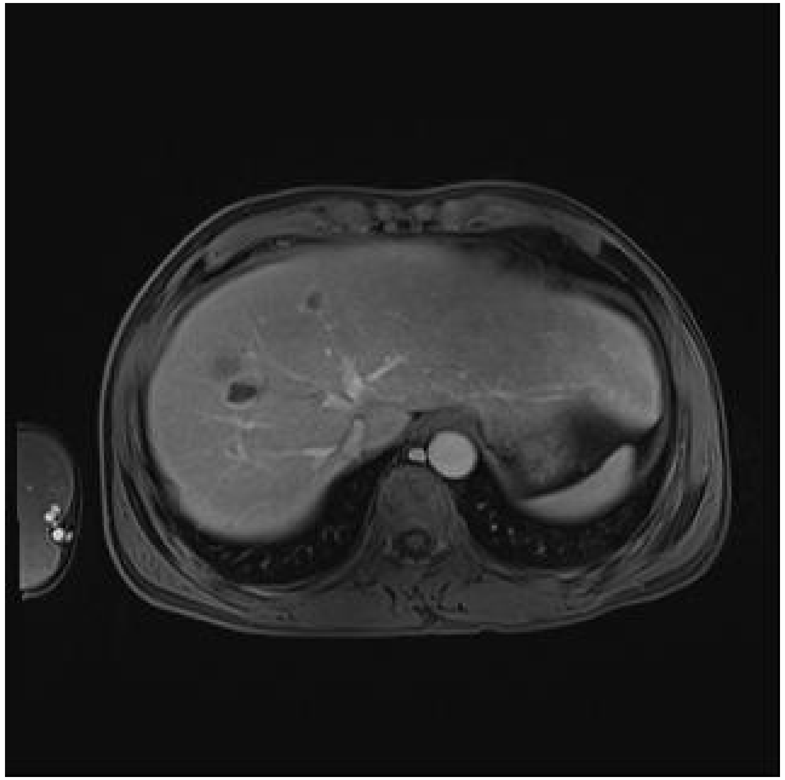

After conversion

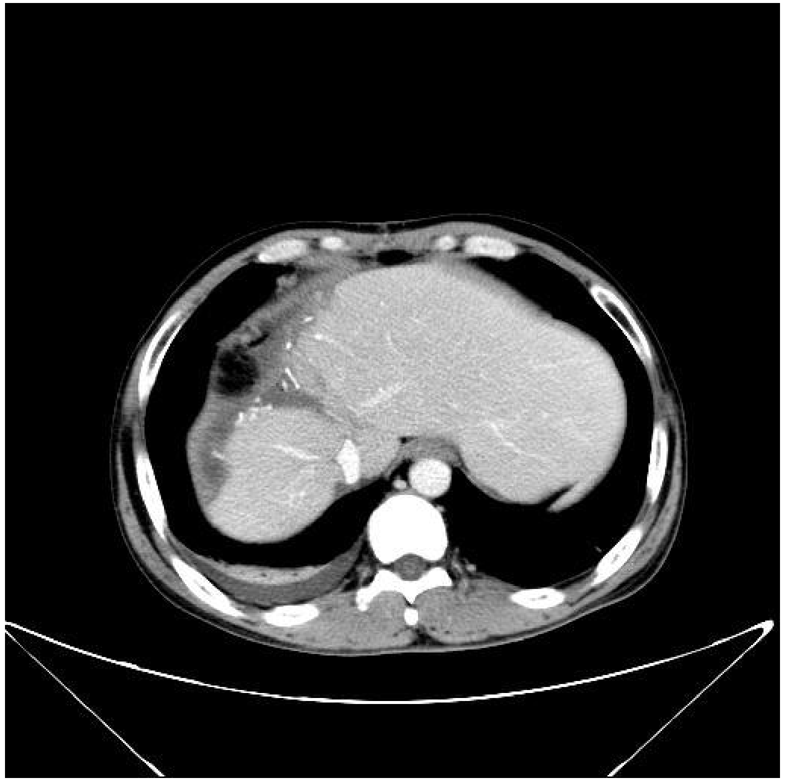

After surgery

Patient 13

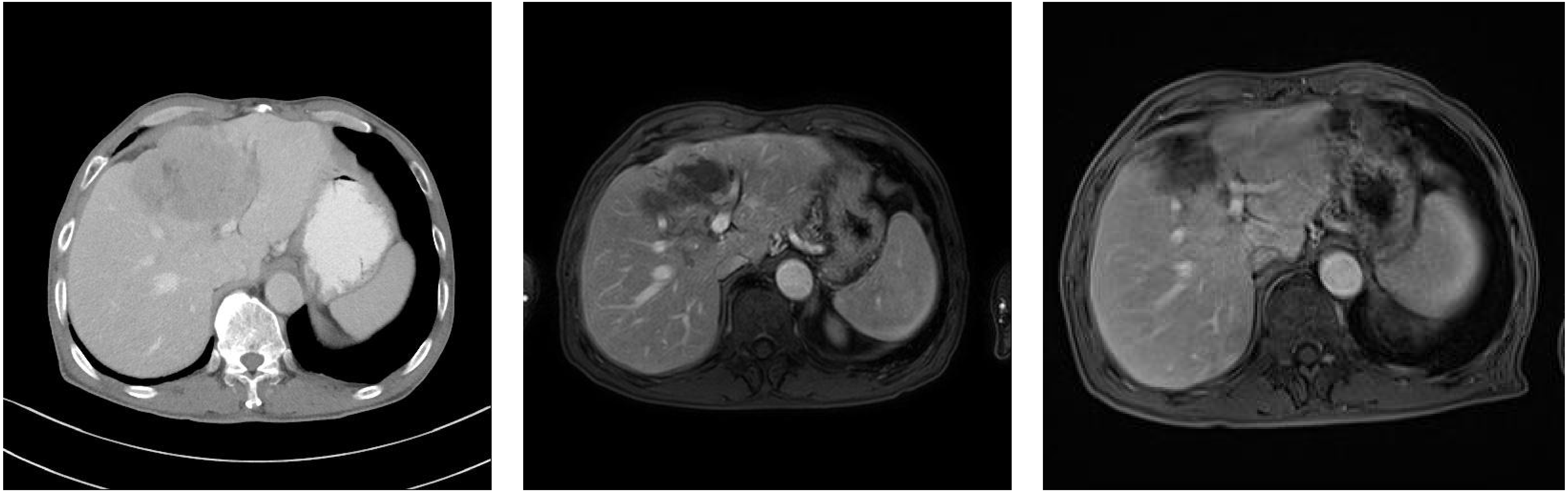

Before conversion After conversion After surgery

Patient 14

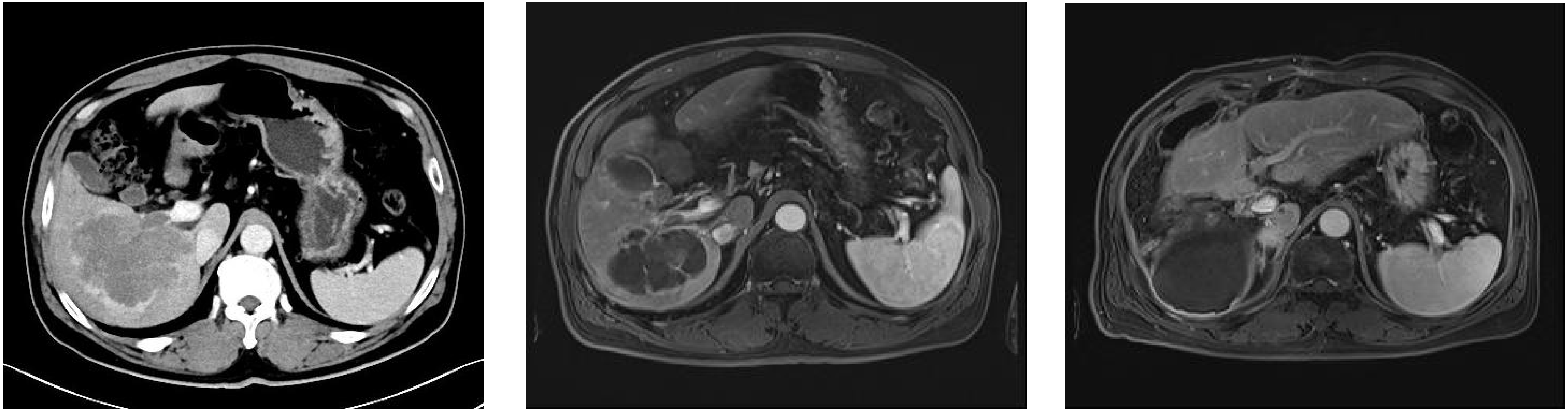

Before conversion | After conversion | After surgery

Patient 15

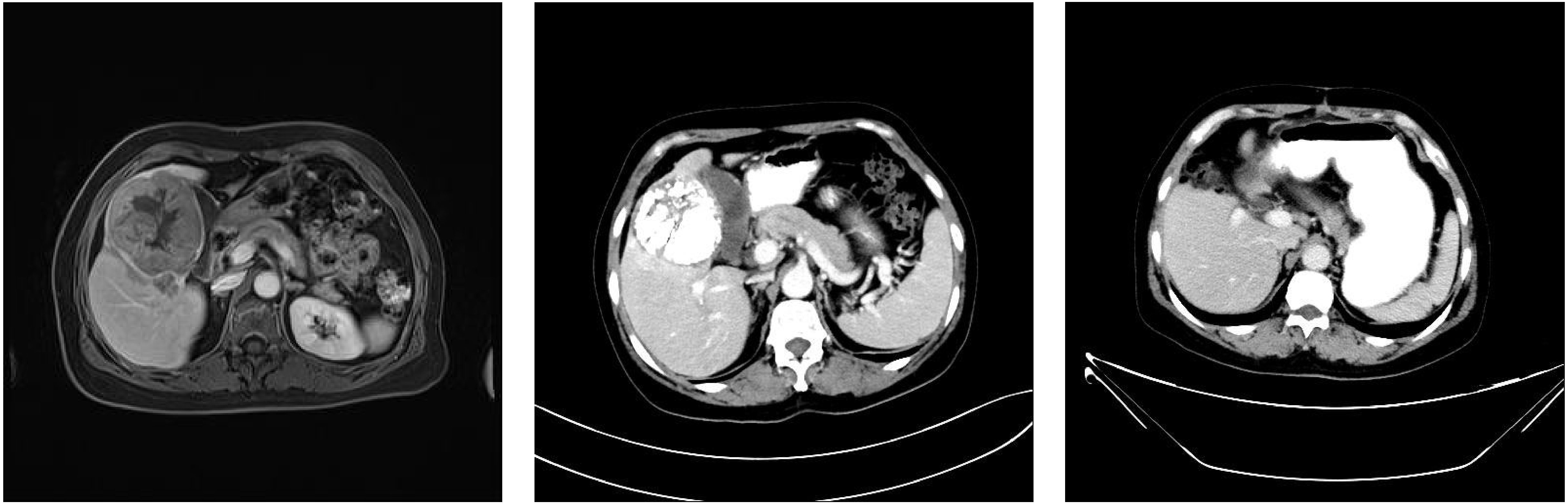

Before conversion After conversion After surgery

Patient 16

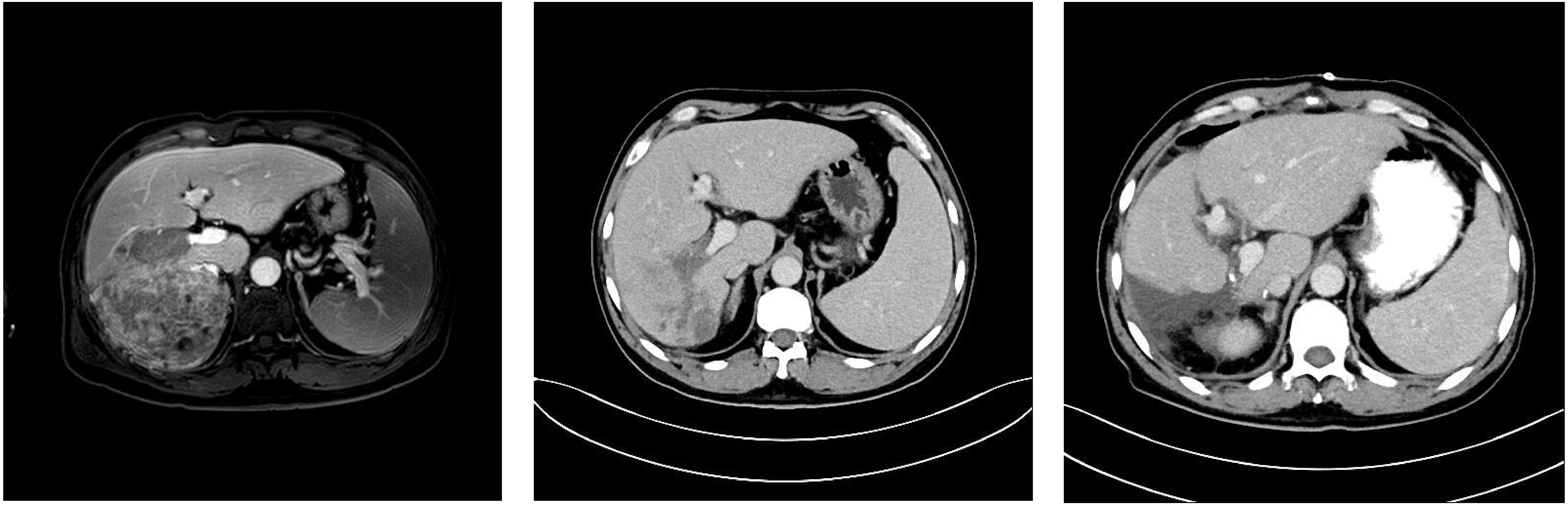

Before conversion | After conversion | After surgery

Patient 17

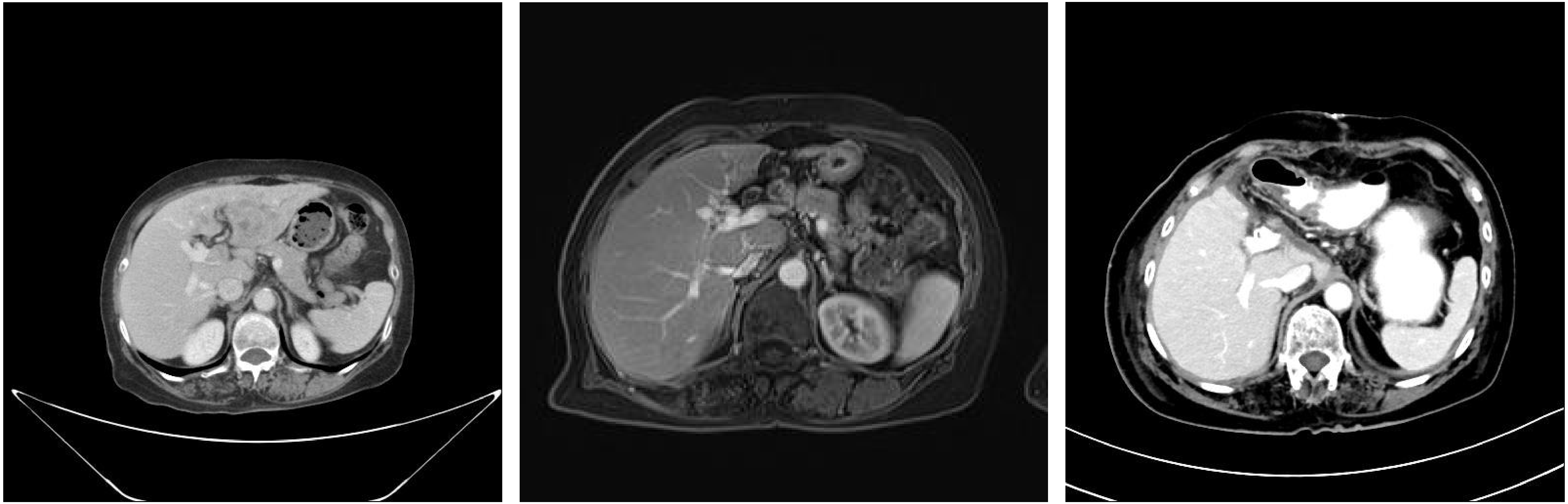

Before conversion   After conversion   After surgery

Patient 18

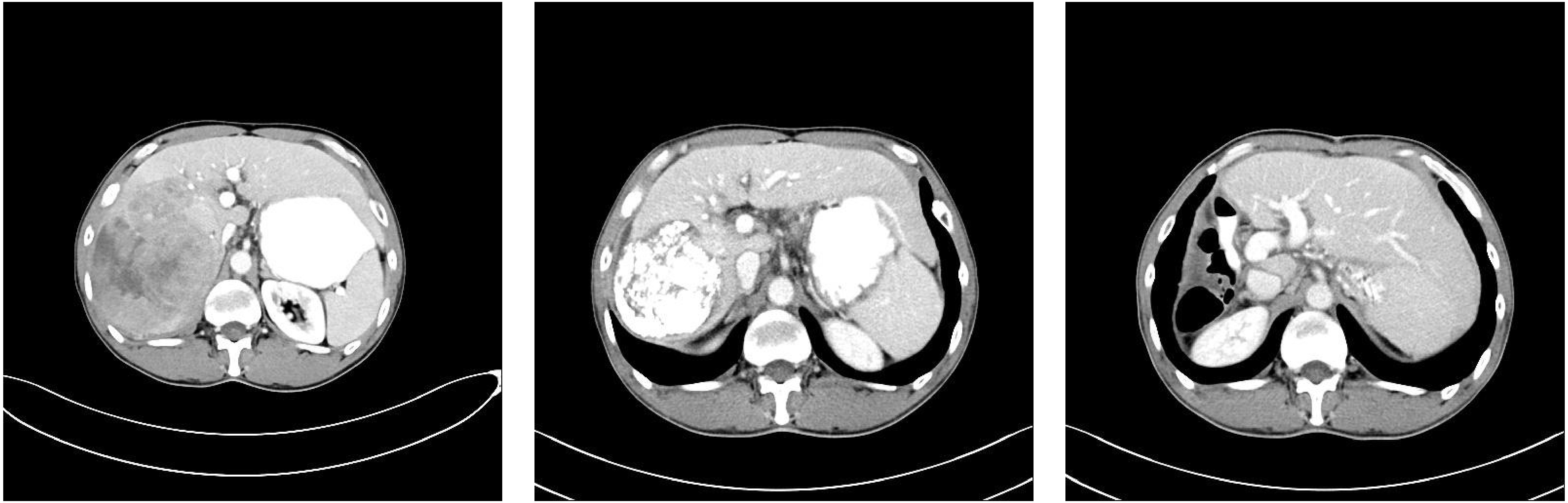

Before conversion

After conversion

After surgery

Patient 19

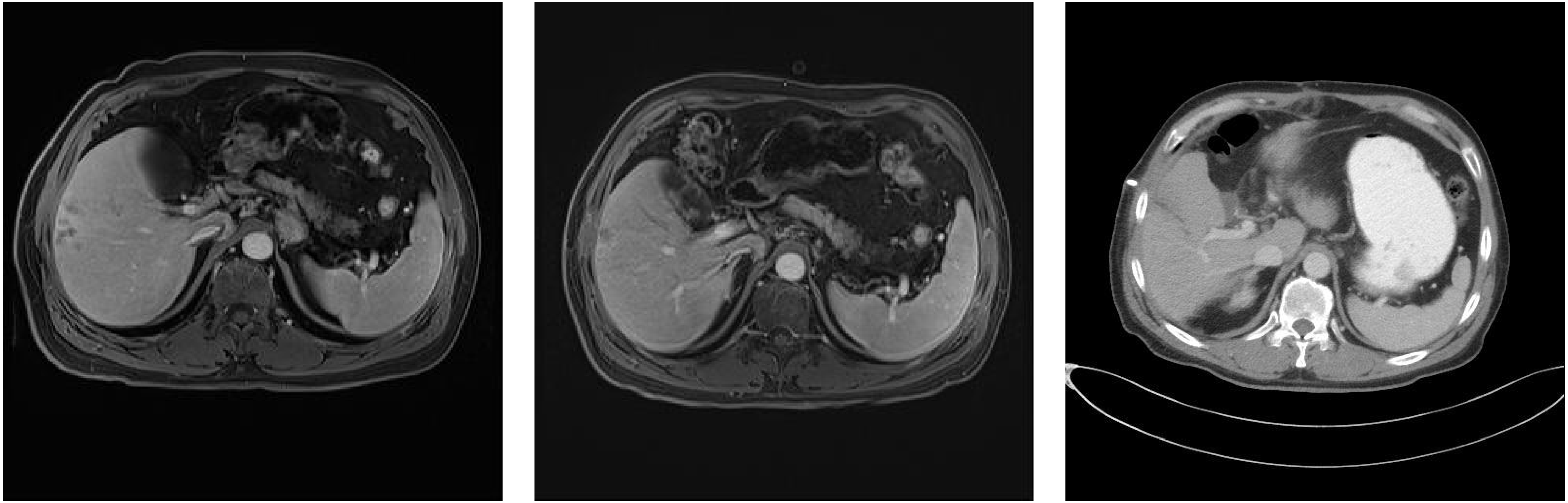

Before conversion After conversion After surgery

Patient 20

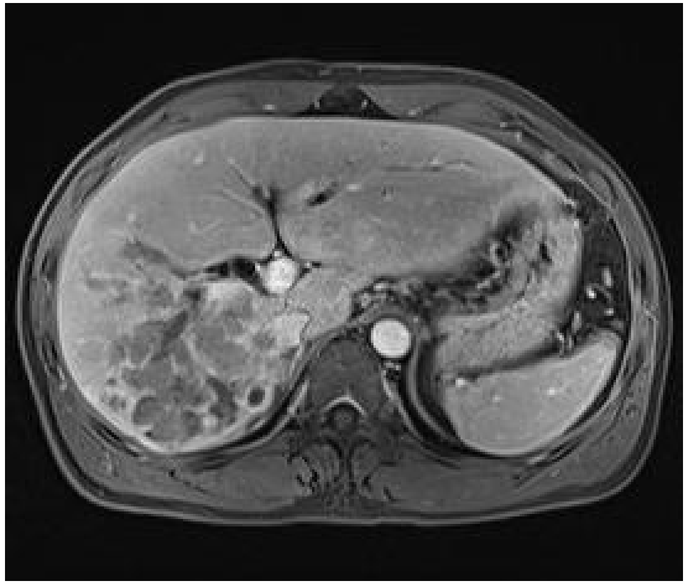

Before conversion

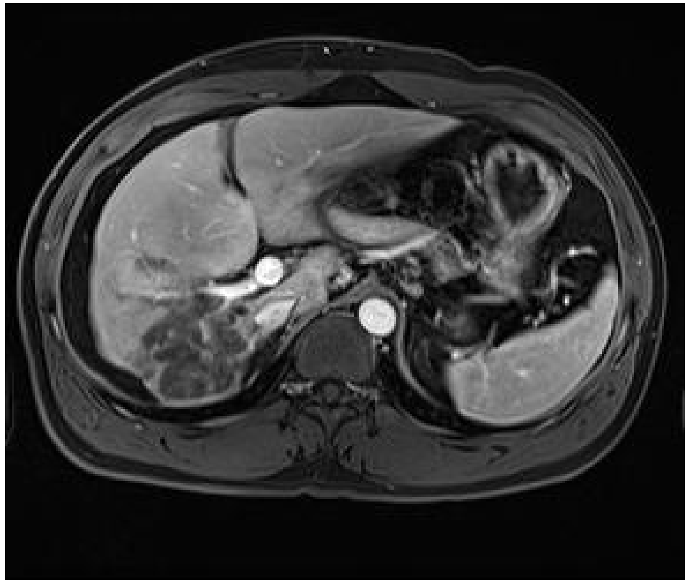

After conversion

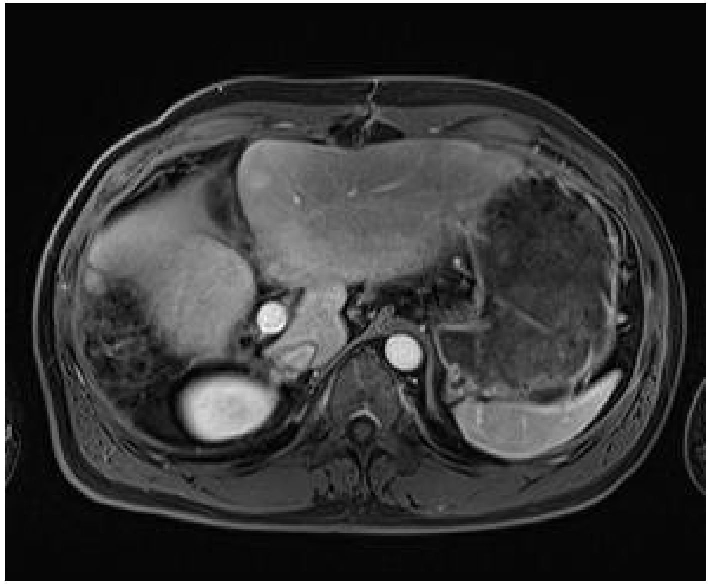

After surgery

Patient 21

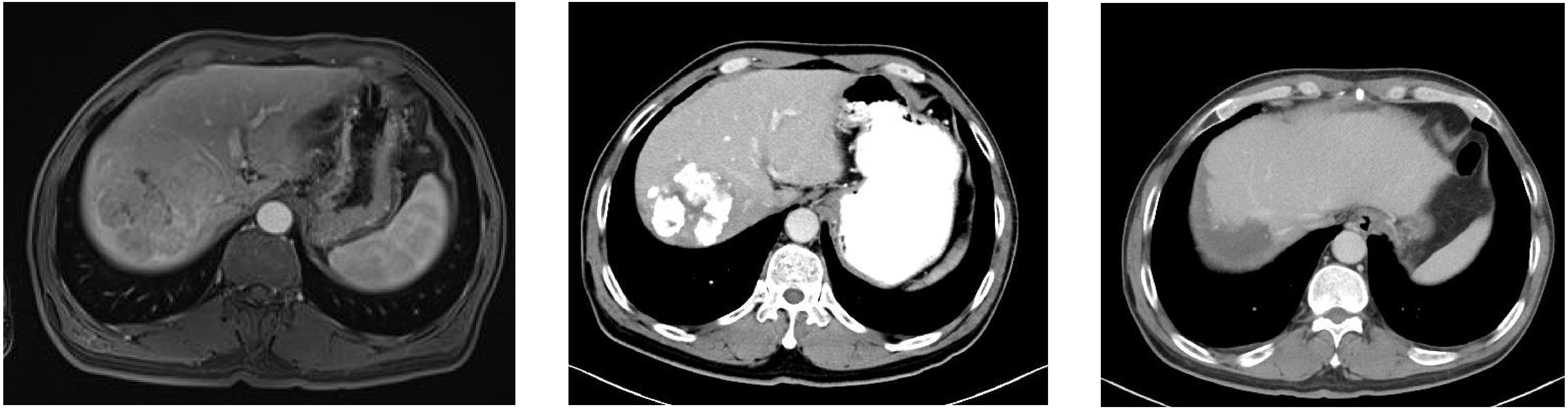

Before conversion After conversion After surgery

Patient 22

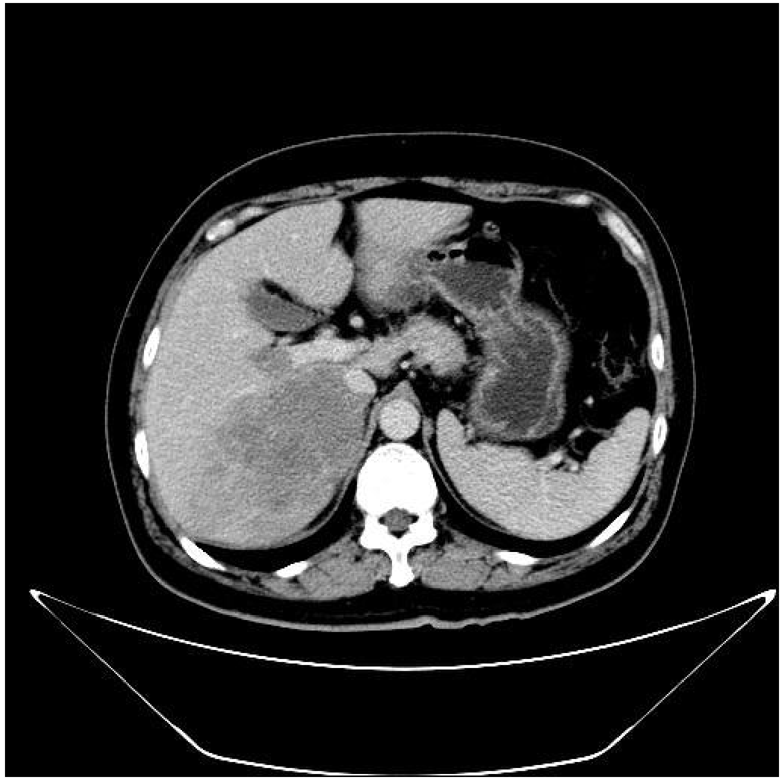
Before conversion

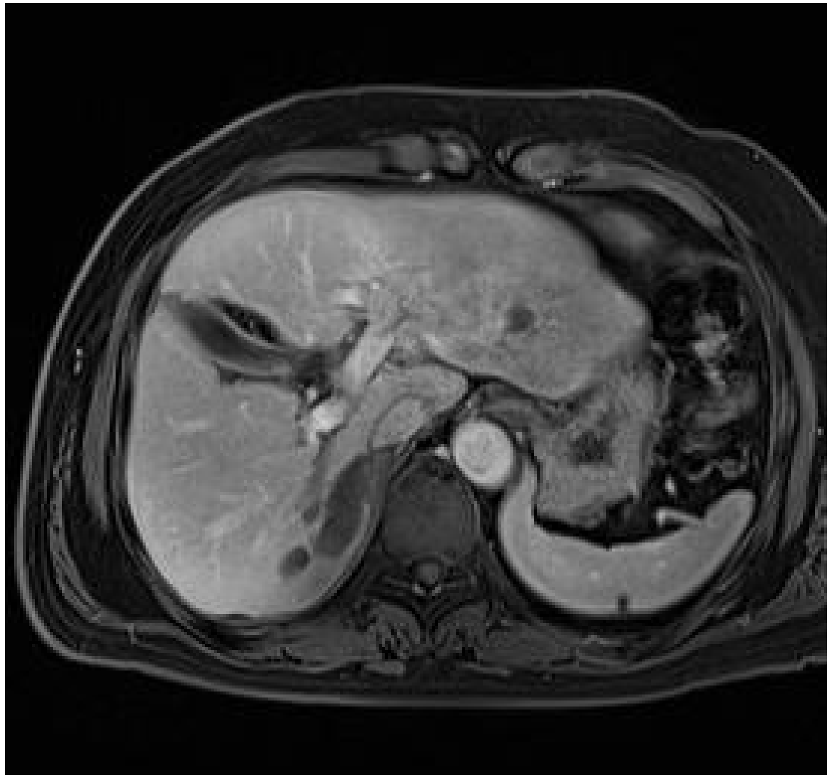
After conversion

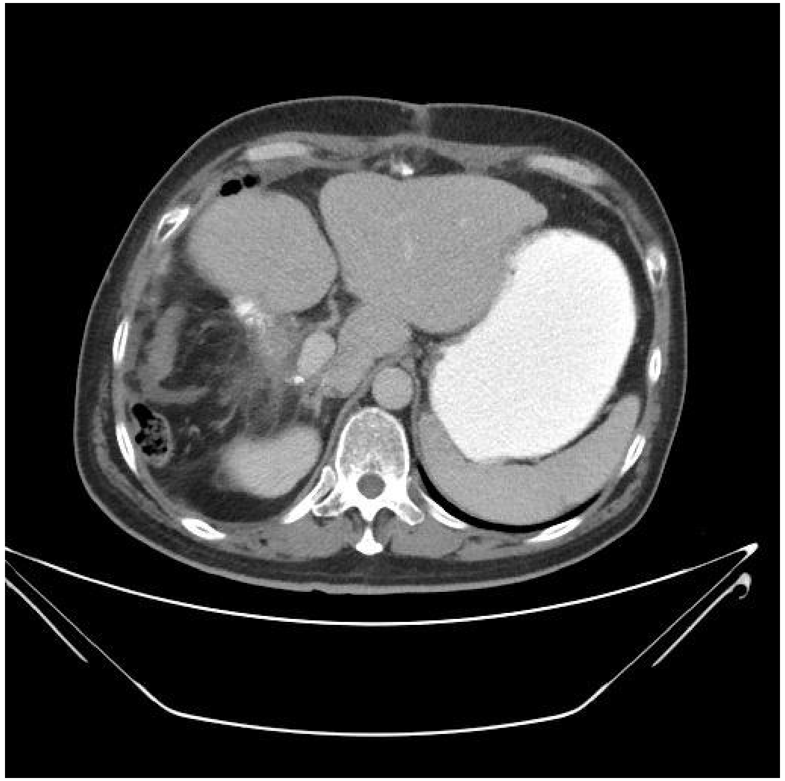
After surgery

Patient 23

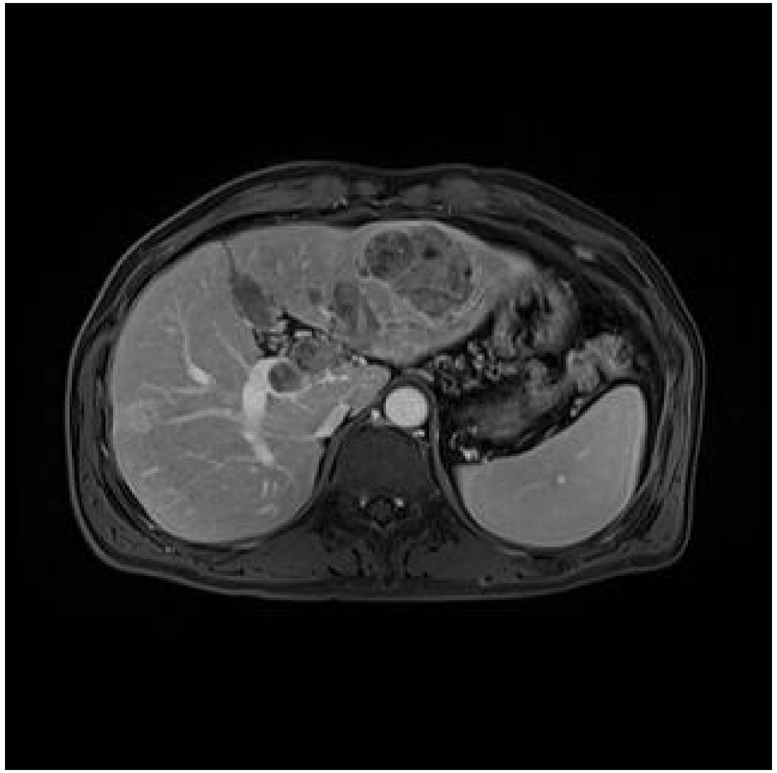

Before conversion

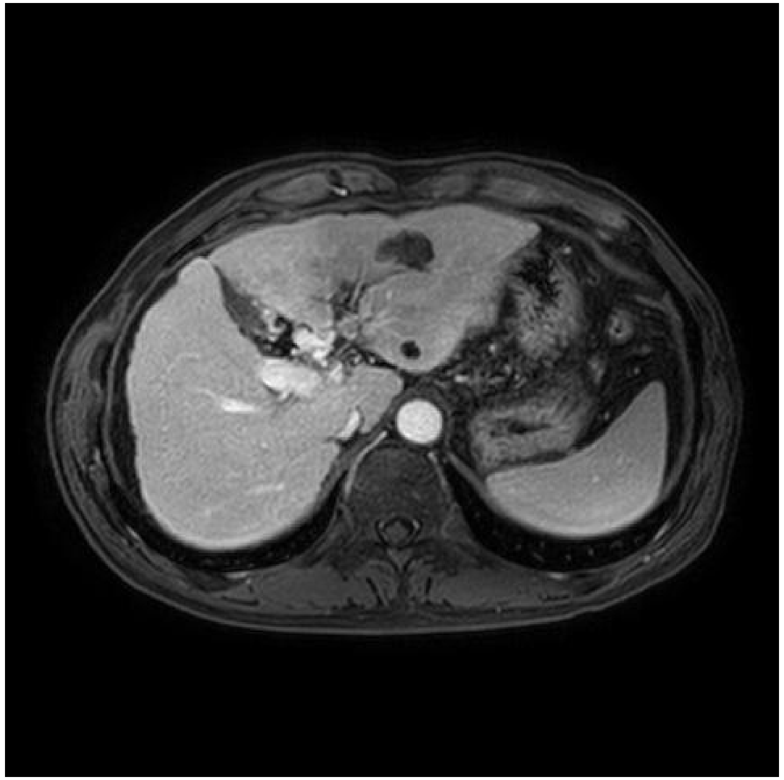

After conversion

Patient 24

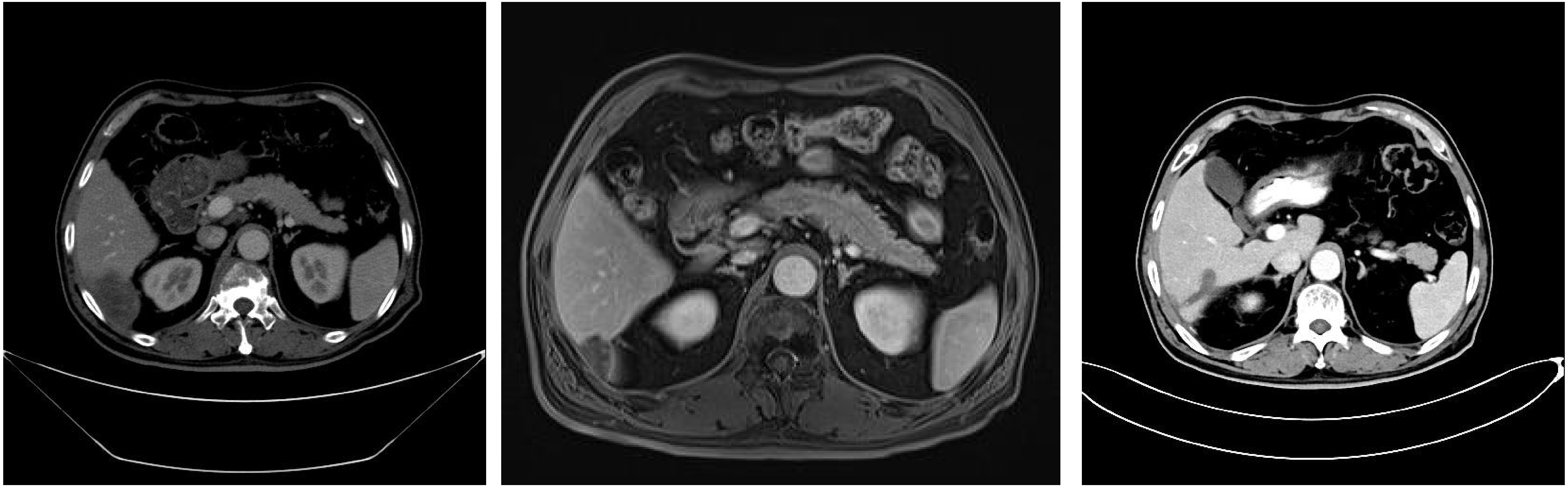

Before conversion　　After conversion　　After surgery

Patient 25

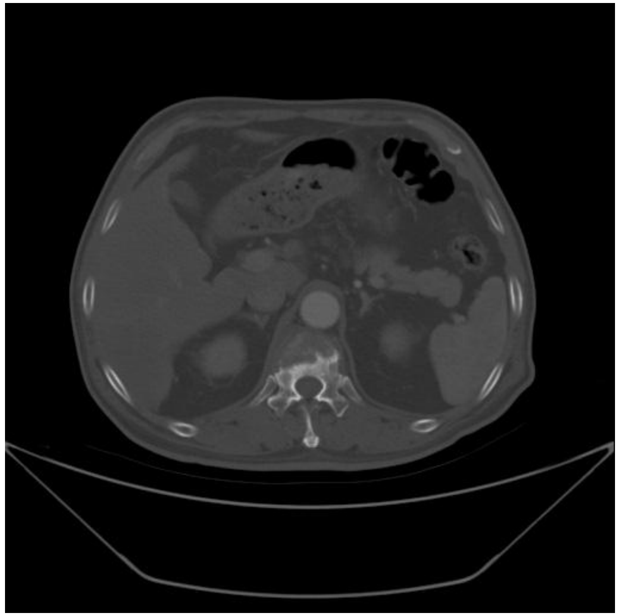

Before conversion

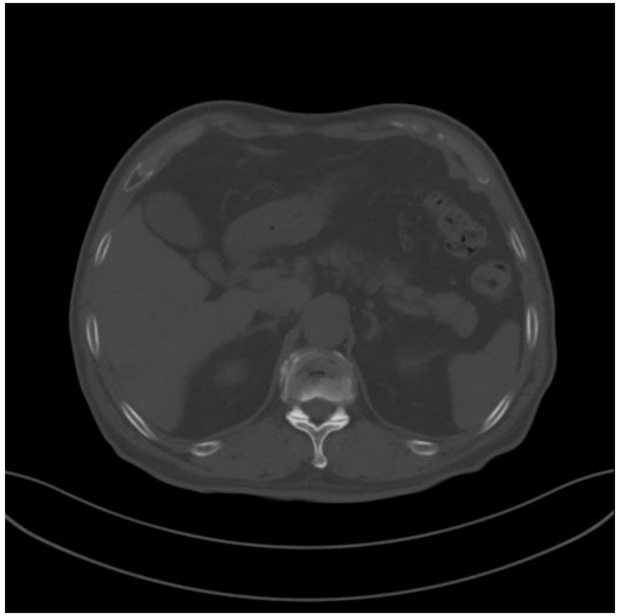

After conversion

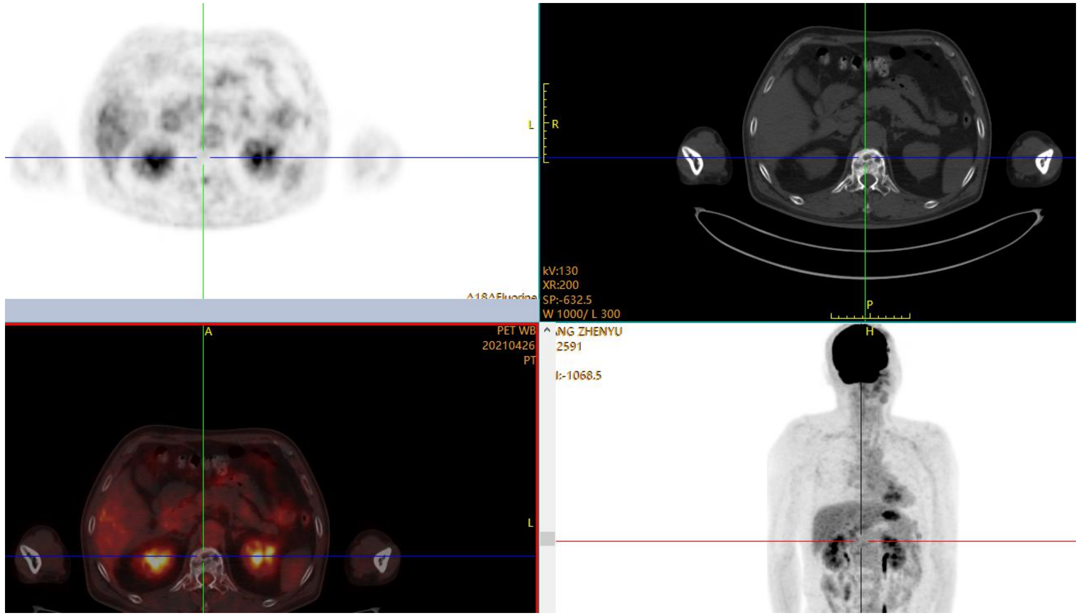

After surgery

Patient 25

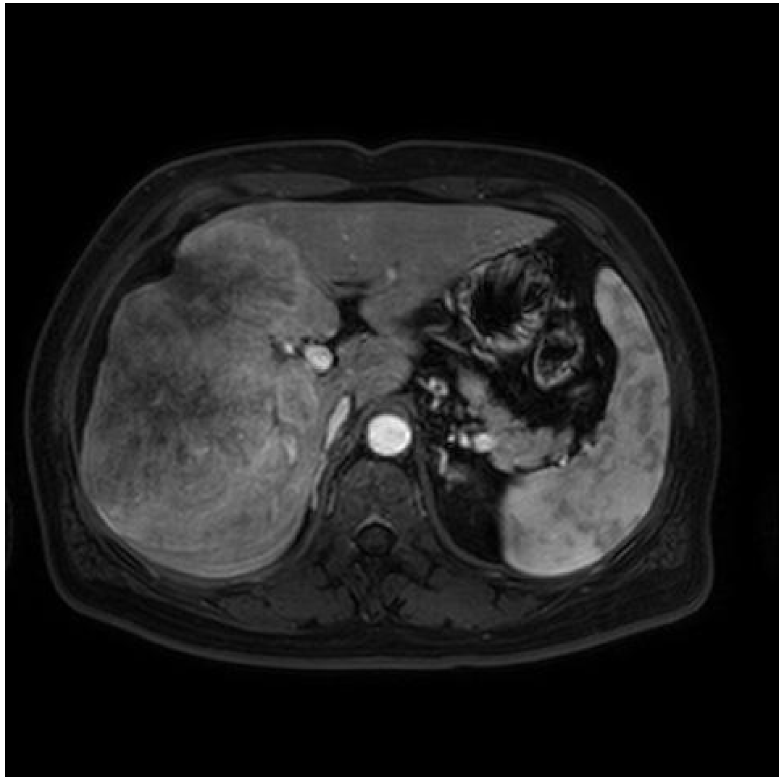

Before conversion

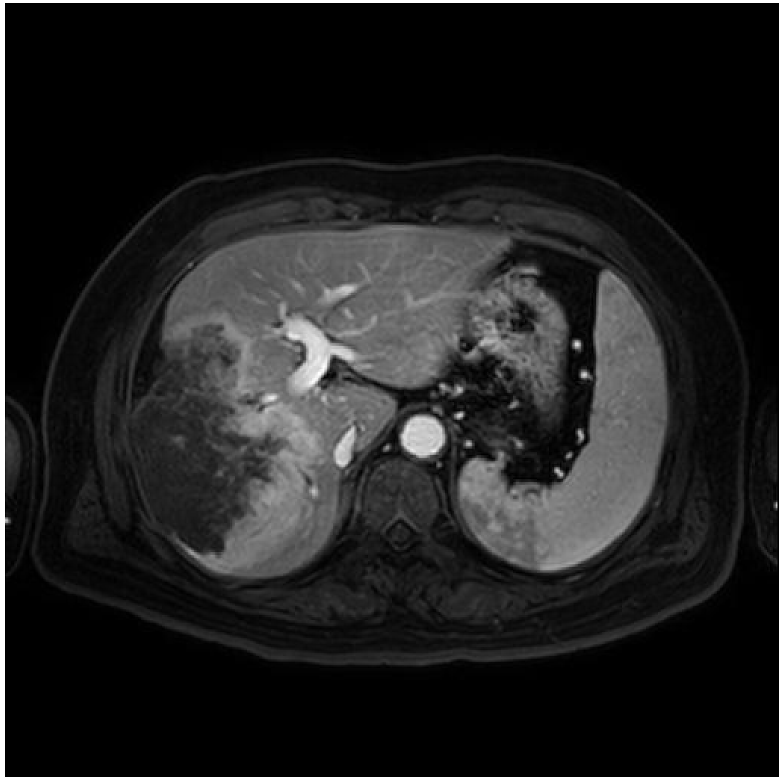

After conversion

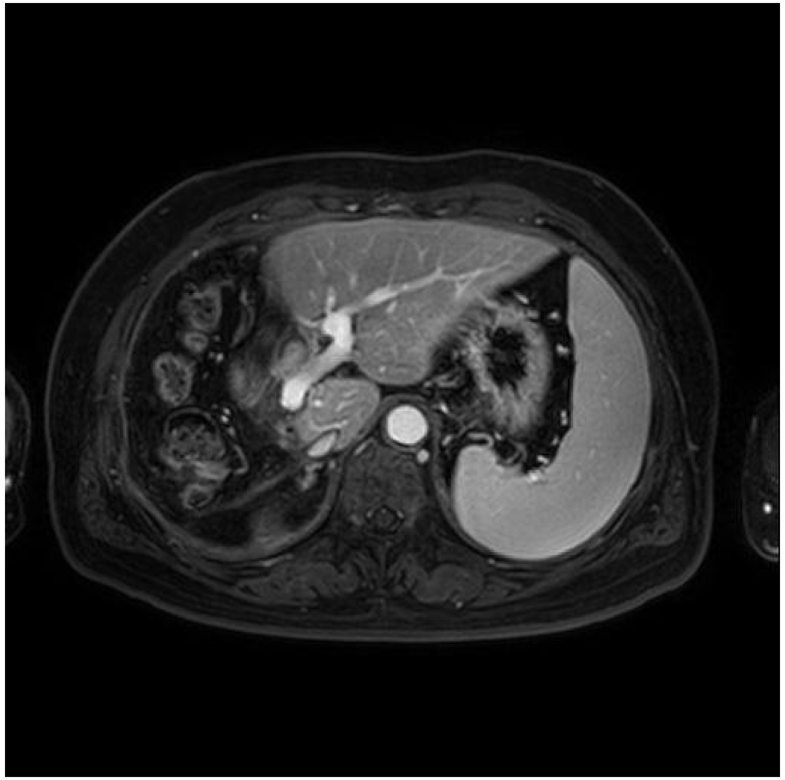

After surgery

Patient 26
